# Discovering correlates of age-related decline in a healthy late-midlife male birth cohort

**DOI:** 10.18632/aging.103345

**Published:** 2020-09-10

**Authors:** Kiyana Zarnani, Stephen M. Smith, Fidel Alfaro-Almagro, Birgitte Fagerlund, Martin Lauritzen, Egill Rostrup, Thomas E. Nichols

**Affiliations:** 1 Functional Imaging Unit, Department of Clinical Physiology, Nuclear Medicine and PET, Copenhagen University Hospital Rigshospitalet, Glostrup, Denmark; 2 Center for Healthy Aging, University of Copenhagen, Copenhagen, Denmark; 3 Department of Neuroscience, University of Copenhagen, Copenhagen, Denmark; 4 Department of Clinical Neurophysiology, Rigshospitalet-Glostrup, Denmark; 5 Center for Neuropsychiatric Schizophrenia Research, Mental Health Center Glostrup, Denmark; 6 Department of Psychology, University of Copenhagen, Copenhagen, Denmark; 7 Wellcome Centre for Integrative Neuroimaging, Oxford Centre for Functional Magnetic Resonance Imaging of the Brain (FMRIB), Nuffield Department of Clinical Neurosciences, University of Oxford, Oxford, UK; 8 Oxford Big Data Institute, Li Ka Shing, Centre For Health Information and Discovery, Nuffield Department of Population Health, University of Oxford, UK; 9 Department of Statistics, University of Warwick, Coventry, UK

**Keywords:** neurocognitive function, brain structure, aging-related decline, magnetic resonance imaging, risk factors

## Abstract

Studies exploring age-related brain and cognitive change have identified substantial heterogeneity among individuals, but the underlying reasons for the differential trajectories remain largely unknown. We investigated cross-sectional and longitudinal associations between brain-imaging phenotypes (IDPs) and cognitive ability, and how these relations may be modified by common risk and protective factors. Participants were recruited from the 1953 Danish Male Birth Cohort (N=123), a longitudinal study of cognitive and brain ageing. Childhood IQ and socio-demographic factors are available for these participants who have been assessed regularly on multiple IDPs and behavioural factors in midlife. Using Pearson correlations and canonical correlation analysis (CCA), we explored the relation between 454 IDPs and 114 behavioural variables. CCA identified a single mode of population covariation coupling cross-subject longitudinal changes in brain structure to changes in cognitive performance and to a range of age-related covariates (r=0.92, P_corrected_ < 0.001). Specifically, this CCA-mode indicated that; decreases in IQ and speed assessed tasks, higher rates of familial myocardial infarct, less physical activity, and poorer mental health are associated with larger decreases in whole brain grey matter and white matter. We found no evidence supporting the role of baseline scores as predictors of impending brain and behavioural change in late-midlife.

## INTRODUCTION

Among the many challenges presented by an increasingly “top heavy” Western society, the cases of cognitive decline and the accompanying economic and social demands have never been more apparent [1, 2]. It is well-established that the ageing brain undergoes major structural and functional changes which, even in the absence of disease, is related to decline in specific cognitive domains [3–9]. Furthermore, it has been shown that on an individual basis, there is significant variability in the trajectories of brain and cognitive change, with a small proportion of the population demonstrating “heathy” or “successful” ageing well into old age [10]. However, the reasons underlying the observed variability is not well-understood [11]. Equally, evidence linking longitudinal brain-cognitive changes to each other and to possible health-related lifestyle behaviours and environmental influencers are limited and inconsistent. Thus, in this present study, our first aim was to explore brain-cognitive longitudinal relations. Our second aim was to identify potential risk and protective factors that may contribute to the individual variations observed in later-life brain structure and cognitive functioning.

In a review of the cumulative research assessing the interdependence of age-related (brain-behaviour) changes in non-pathological conditions, the findings are unclear [9]. Although evidence of coupled changes are commonly described in the direction of advancing age, less intact brain structures, greater brain structural degradation and lower cognitive ability [12–14], associations that oppose this common course of age-related change are also reported [15, 16]. Examples include correlations that link larger initial brain volume and greater negative (shrinkage) change [17], higher early-life IQ and greater decline in visuospatial abilities [18], higher crystallized abilities and greater brain volume reduction and thinning of the cerebral cortex [19], smaller baseline regional brain volume and a moderate (gradual) rate of decline [20]. The varying and at times contradictory findings have been attributed to many factors namely, variations in sample characteristics, small sample size, short observation intervals, inclusion of subjects with undiagnosed pathology, modest within-subject change and between-subject differences in change.

The majority of studies exploring age-related changes in brain and cognition use population-based cross-sectional samples [21]. Such studies largely converge onto several common trends that link increasing age to whole-brain and regional atrophy [22–25], increases in ventricular volume [26], and the accumulation of neural insults of cerebrovascular origin [27–30]. Furthermore, the age-brain effects observed indicate regional specificity that broadly describe an anterior-to-posterior gradient of decline with frontal, temporal and posterior association cortices appearing most vulnerable, and the brainstem, pons and primary sensory cortex showing negligible, if any, age-related variability [8, 17, 20, 31–38]. Similarly, age-effects on cognitive performance also suggest an underlying preservation of specific cognitive domains, with fluid abilities (e.g. processing speed, executive functioning, working memory, and inhibitory functions) appearing most vulnerable to increasing age, and crystallized abilities (e.g. general knowledge, implicit procedural long-term memory, numerical processing) appearing relatively spared [2, 6, 8, 14, 18, 39–42].

However, as the study of ageing is fundamentally the study of change, the use of cross-sectional data based on single observations from individuals of different ages is not an ideal study design. Specifically, cross-sectional studies can only offer information on age-related individual differences in level, and not individual differences in change. The problem here is one of aggregation in so far as pooling data across age-groups may result in misleading “illusory associations” that are in fact based on average age differences (i.e. an example of Simpsons Paradox and Lord’s paradox [43, 44]). Thus, although ideal for estimating population-level mean trends [37, 45], cross-sectional samples are ill-equipped in providing reliable estimates of intra-individual change and the associations between rates of change. Adding to this, cross-sectional studies that span a wide age-range are also highly vulnerable to cohort effects, secular trends, and any other overlooked individual differences that are brought into the study from previous years. Considering this, the ability to measure within subject changes independently of between subject differences demands that the same individual is followed over time using a longitudinal design. Although findings among longitudinal studies are generally more consistent than their cross-sectional counterparts’ [21], they are also bound by their own limitations such as the ‘3M’ – mobility, morbidity and mortality of subjects [46]. Notably, as many longitudinal studies start off as cross-sectional samples that are based on age-heterogeneous groups there is still a risk of mixing individual differences in rates of change (i.e. random age effects) with average age-dependent changes at the population level (i.e. fixed age effects) [37].

Lastly, many studies investigating potential correlates of age-related changes typically include a small number of putative risk and protective factors. Due to mutual-interrelations, such studies are at increased risk of identifying relations that are, in part, or entirely confounded by variables that have been overlooked. Of the studies that do include a wide range of potential age-related modifiers, it is rare that their affects are examined simultaneously. Specifically, the application of improper statistical models to essentially explore the same ageing-related hypotheses, may be largely accountable for the inconsistent results observed across studies. Thus, studies using a narrow-age longitudinal sample, a large multidimensional dataset, and multi-level statistical modelling can reduce many of the aforementioned types of confounding discussed in order to increase the precision of estimated effects. Additionally, compared to focused analyses that explore the relation between specialized anatomical regions and specific cognitive tests, a multifactorial approach is ideal for revealing relations that may have so far been overlooked in ageing research.

Thus, in this present study, we use a single-year-of-birth cohort where the majority of subjects have completed two early intelligence quotient (IQ) tests at ages ~11 (IQ-11) and ~20 (IQ-20). Subsequent, brain-imaging and behavioural assessments were conducted in two late-midlife waves separated by an observation interval of ~5 years. Here, detailed neuropsychological, brain MRI, general health, demographic, and lifestyle data have been acquired to investigate three key questions: First, exploring cross-sectional-longitudinal associations we asked: do midlife-baseline (age ~57; W-57) and follow-up (age ~63; W-63) brain structure correlate with changes in cognitive functioning, and relatedly, does W-57 or W-63 cognitive ability correlate with changes in brain structure? Second, we explored the impact of pure cross-sectional information on longitudinal associations: How are associations of longitudinal change in brain structure and cognitive ability altered when average measures are controlled for (i.e., assessed by comparing correlations between longitudinal changes before and after regressing out average measures ((W-57+W-63)/2)? Third, we explored the extent and direction in which common age-related risk and protective factors influence the observed brain-cognition relations in questions 1 and 2.

## RESULTS

Participant characteristics are reported in Tables 1–5. Longitudinal change in cognitive ability and brain imaging structural measures are shown in Supplementary Figures 1, 2.

**Table 1:** List and study sample characteristics of cognitive measures acquired at W-57 and W-63.

**COGNITIVE MEASURES (N=31)**					
	**TEST**	**MEAN**		**SD**	
**General intelligence**	Härnquist (IQ-11)	74.14		15.72	
	BP (IQ-18)	45.68		8.73	
	IST2000-R (IQ-57)	32.47		12.69	
	IST2000-R (IQ-63)	31.46		10.80	
**DOMAIN**	**CANTAB**	**MEAN**	**SD**	**MEAN**	**SD**
		**W-57**		**W-63**	
**Visual paired associates learning and memory**	**Paired Associates Learning (PAL)**				
	First trial memory score	17.24	3.36	18.65	3.20
	Total Errors Adjusted	21.44	20.99	16.39	16.44
	Total Trials Adjusted	14.23	4.21	13.34	3.47
**Pattern recognition memory**	**Pattern Recognition Memory (PRM)**				
	Percent correct	89.90	8.54	90.83	8.03
	Standard deviation correct latency (msec)	883.51	526.19	714.95	401.79
**Spatial recognition memory**	**Spatial Recognition Memory (SRM)**				
	Percent correct	83.74	8.11	82.58	8.73
	Standard deviation correct latency (msec)	1478.51	692.21	1209.45	700.68
**Motor skills**	**Motor Screening (MOT)**				
	Mean Error	9.11	2.09	6.29	1.70
	Mean Latency (msec)	1104.22	368.91	877.62	192.78
**Reaction time**	**Reaction Time (RTI)**				
	Mean 5-choice movement time	380.74	92.52	389.73	96.30
	Mean 5-choice reaction time	365.20	45.78	362.00	64.32
**Attention**	**Rapid Visual Processing (RVP)**				
	A’ Score	0.91	0.05	0.91	0.10
	Mean latency block 1 (msec)	382.09	134.56	386.87	146.71
	Mean latency block 2 (msec)	341.52	120.53	330.29	122.16
	Mean latency block 3 (msec)	354.82	113.66	327.78	81.81
	Mean latency block 4 (msec)	419.46	104.04	416.42	141.03
**Global cognitive functioning**	**ACE**				
	Total Score	94.23	4.22	93.25	5.33
	**MMSE**				
	Total Score	29.29	0.96	29.15	1.16
**Executive function (planning)**	**Stockings of Cambridge (SOC)**				
	Problems solved in minimum moves	9.45	1.43	9.15	1.65
	Mean 5-moves	5.93	0.98	6.26	1.13
	Mean initial thinking time 5-moves (sec)	15.69	14.39	10.76	7.45
	Mean subsequent thinking time 5-moves (sec)	1.54	2.86	0.55	0.84
**DOMAIN**	**PAPER AND PENCIL**	**MEAN**	**SD**	**MEAN**	**SD**
**Verbal paired associative learning and memory**	**15 Word Pairs Recall and Retention**				
	Learning	11.15	8.39	12.52	9.39
	Retention	4.52	3.46	5.01	3.68
**Processing speed**	Digit symbol modalities test	47.99	8.09	47.50	8.65
	Trail Making A (sec)	33.08	9.68	30.51	8.14
	Trail Making B (sec)	73.99	28.77	70.42	20.45

Abbreviations: BP = Børge Priens Test; IST = Intelligenz-Struktur-Test 2000 R; CANTAB = Cambridge Neuropsychological Test Automated Battery; ACE = Addenbrooke’s Cognitive State Examination; MMSE = Mini Mental State Examination). Total cognitive measures included n=31.

**Table 2:** List and study sample characteristics of social and biological demographic measures acquired at W63.

**DEMOGRAPHIC MEASURES (N=8)**	
	W63
**Social**	**N (%)**
	
**Subject SEP**	
Working	95 (77.2%)
Early retirement	4 (3.3%)
In education	1 (0.8%)
Without/unknown	23 (18.7%)
**Paternal SEP**	
Self-employed, employee, or civil servant	57 (46.3%)
Skilled worker	30 (24.4%)
Unskilled worker	26(21.1%)
Without/unknown	10 (8.1%)
**Education Attainment**	
University	25 (20.3%)
Vocational	33 (26.8%)
High School/A-levels	46 (37.4%)
Unskilled	4(3.3%)
Without/unknown	15 (12.1%)
**Civil Status**	
Single (no)	89 (72.4%)
Single (yes)	11 (8.9%)
Unknown	23 (18.6%)
**Offspring**	
No	12 (9.7%)
Yes	88 (71.5%)
Unknown	23 (18.7%)
**School Years**	
Mean (SD)	10.9 (2.32)
	
**Biological**	**MEAN (SD)**
Birth length (cm)	52.22 (2.83)
Birth weight (kg)	3.51 (0.47)

Abbreviations: SEP = social economic position; SD = standard deviation, g =grams. Total demographic measures included n=8.

**Table 3:** List and study sample characteristics of health measures acquired at W57 and W63.

**HEALTH MEASURES (N=25)**			
**Prevalence of NCDs (self-reported)**	**YES (%)**	**NO (%)**	**Unknown (%)**
Asthma	5 (4.1%)	101 (82.1%)	17 (13.8%)
Cancer	3 (2.4%)	103 (83.7%)	17 (13.8%)
Cardiovascular	17 (13.8%)	89 (72.4%)	17 (13.8%)
Cerebrovascular	11 (8.9%)	95 (77.2%)	17 (13.8%)
Depression	6 (4.8%)	100 (81.3%)	17 (13.8%)
Diabetes	5 (4.1%)	101 (82.1%)	17 (13.8%)
Hypercholesterolemia	14 (11.4%)	92 (74.8%)	17 (13.8%)
Hypertension	36 (29.3%)	70 (56.9%)	17 (13.8%)
Migraine	9 (7.3%)	97 (78.9%)	17 (13.8%)
Prolapsed Disc	6 (4.8%)	100 (81.3%)	17 (13.8%)
**Prevalence of familial history of NCDs (self-reported)**			
Cardiovascular	24 (19.5%)	75 (60.9%)	24 (19.5%)
Cerebrovascular	23 (18.7%)	83 (67.5%)	17 (13.8%)
Dementia	29 (23.6%)	70 (56.9%)	24 (19.5%)
Diabetes	20 (16.3%)	79 (64.2%)	24 (19.5%)
Depression	27 (22.0%)	73 (59.3%)	23 (18.7%)
Hypertension	34 (27.6%)	60 (48.8%)	29 (23.6%)
Myocardial Infarct	20 (16.3%)	79 (64.2%)	24 (19.5%)
**Common health biomarkers (MEAN, SD)**	**W57**	**W63**	
BMI (kg/m2)	26.46 (3.21)	24.86 (8.56)	-
Total Cholesterol mmol/L	5.44 (1.38)	5.14 (1.14)	-
HDL Cholesterol mmol/L	1.38 (0.41)	1.02 (0.36)	-
LDL Cholesteol mmol/L	3.44 (1.14)	3.01 (1.13)	-
VLDL Cholesterol mmol/L	0.61 (0.32)	0.63 (0.33)	-
Triglyceride Cholesterol mmol/L	1.41 (0.88)	1.54 (1.00)	-
Major Depression Inventory (MDI) score	-	4.39 (4.07)	-
Cerebral Blood Flow* (mL/min)	52.82 (12.13)	-	-

Abbreviations: NCDs = non-communicable diseases; BMI = body mass index; SD = standard deviation. Total health measures included n=25. *Cerebral blood flow is normalised to brain size.

**Table 4:** List and study sample characteristics of lifestyle variables acquired at W63.

**LIFESTYLE MEASURES (N=9)**	
**Alcohol**	**N (%)**
Status	
Yes	100 (81.3%)
No	3 (2.4%)
Unknown	20 (16.3%)
	**MEAN (SD)**
Start age	15.20 (1.75)
Units per week	10.39 (12.44)
**Exercise (frequency)**	**N (%)**
Daily	25 (20.3%)
2-3 per week	40 (32.5%)
1 per week	12 (9.7%)
2-3 per month	4 (3.2%)
Few per year	6 (4.9%)
Never	12 (9.76%)
Unknown	24 (19.5%)
**Smoking**	**N (%)**
Status (yes)	65 (52.8%)
Status (no)	35 (28.5)
Unknown	23 (18.7%)
	**MEAN (SD)**
No of smokes per day	9.14 (11.7)
Age start (years)	15.19 (3.36)
Age stop (years)	39.56 (11.55)
**Sleep quality**	**MEAN (SD)**
Pittsburgh Sleep Quality Index (PSQI)	4.26 (2.32)

Abbreviations: SD = standard deviation. Total lifestyle measures included n=9.

**Table 5:** List and study sample characteristics of image-derived phenotypes (IDP) acquired at W-57 and W-63.

**Total brain volumes, volumes of individual brain tissues, and diffusion indices at W-57 and W-63**		
	**W-57**	**W-63**
**Image-derived Phenotype (IDPs)**	**Mean (SD)**	**Mean (SD)**
Total brain volume (mm^3^)	1474870 (63818)	1439169 (65578)
Grey matter volume (mm^3^)	752782 (35367)	748454 (36162)
White matter volume (mm^3^)	722088 (41990)	690714 (41104)
WMH volume (normalized)	2.57 (0.06)	3.21 (0.43)
FA (normalized 0-1)	0.60 (0.08)	0.59 (0.08)
MD	0.0007 (0.0001)	0.0007 (0.0001)
MO	0.61 (0.14)	0.61 (0.13)
L1-L3	0.0007 (0.0004)	0.0007 (0.0004)

Total mean brain (grey matter and white matter) volume, total grey matter volume of ROIs and subcortical brain structures, total macrostructural white matter volume, total volume of WMH, and microstructural properties of specific WM tracts pertaining to W-57 and W-63. Diffusion tensor indices (FA, MD, MO, L1-L3) are based on eigenvalues (λ1,λ2,λ3) which describe the magnitude of diffusion within a voxel. Abbreviations: SD = standard deviation, WMH = white matter hyperintensity, FA = fractional anisotropy, MD = mean diffusivity, MO = mode tensor, L1-L3 = eigenvalues).

### Univariate analyses

### Cross-sectional-longitudinal and longitudinal correlations

**Figure 1:**
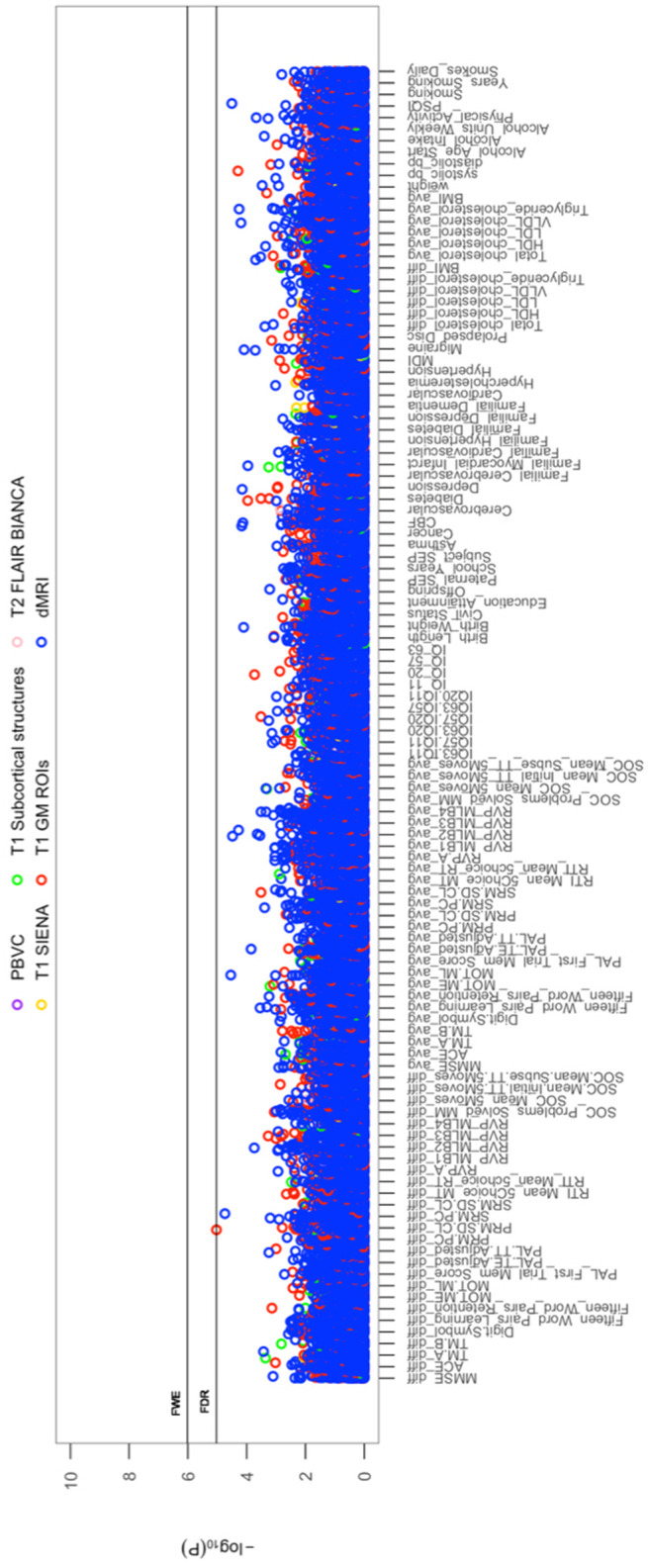
**The significance of associations between IDP and behavioural variables, for longitudinal correlations.** The Manhattan plot shows all results for 454 IDPs against each of the 114 behavioural (51,756 values) adjusted for confounders: age, motion, and head size. Significance is plotted as -log _10_ p-values, arranged by behavioural measures on the x-axis, multiple testing thresholds across all pairwise associations are marked with a horizontal line (FWE: 6.01 x 10^-6^; FDR: 5.03 x 10^-5^). IDPs are distinguished by plotting colour, determined by MRI modality and image processing tool applied to derive each measure. This created 6 imaging subdomains: 1) T1w percentage brain volume change (PBVC) modelled by SIENA 2) T1w global brain volume measures (normalized and unnormalized for head size) modelled by SIENAX (yellow), 3) T1w subcortical structures (shapes and volumes) modelled by FIRST (green), 4) T1w total grey matter volume within grey matter region-of-interests using partial volume estimates derived from FAST (red), 5) T2w-FLAIR total volume of white matter hyperintensities modelled by BIANCA (pink), 6) dMRI estimates of diffusivity measures contained within 48 standard-space WM tract region-of-interests modelled by TBSS (blue). Abbreviations: IQ-11, IQ-20, IQ-57, IQ-63 = general intelligence score ages ~11, ~20, ~57, and ~63; MOT = motor task; ME = mean error; ML = mean latency; PAL = paired associates learning; TE adjusted = total errors adjusted; TT Adjusted = total trials adjusted; PRM = pattern recognition memory; SD = standard deviation; CL = correct latency; RTI = reaction time task; MT = movement time; RT = reaction time; RVP = rapid visual processing task; MLB1-4 = mean latency block 1 to 4; SOC = Stockings of Cambridge; Mean Initial TT 5 Moves = mean initial total time 5 moves task; Mean Subse TT 5 Moves = mean subsequent thinking time 5 moves task; SRM = spatial recognition memory; TM = trail making task; SEP = social economic position; MDI = Major Depression Inventory; CBF = cerebral blood flow; PSQI = Pittsburgh Sleep Quality Index.

**Figure 2:**
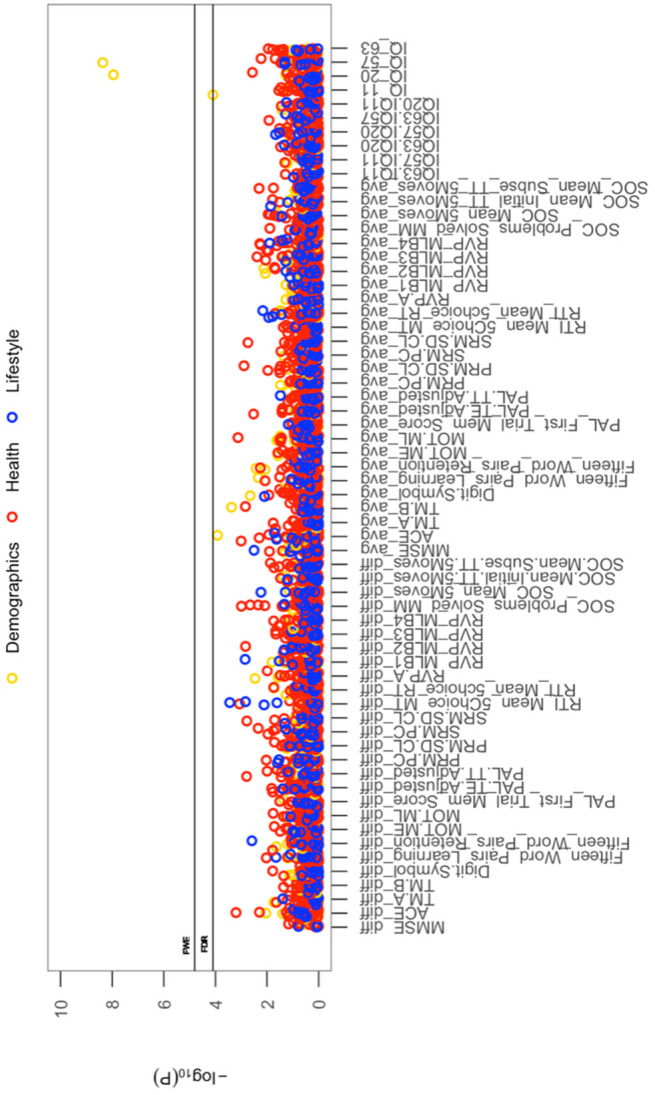
**The significance of associations between each longitudinal/average cognitive measure and all (other) cross-sectional behavioural variables.** The Manhattan plot shows all results for 64 cognitive variables against each of the 50 (other) behavioural variables (3200 values) adjusted for confounders: age, motion, and head size. Significance is plotted as -log _10_ p-values, arranged by cognitive variables on the x-axis, multiple testing thresholds across all pairwise associations are marked with a horizontal line, FWE top line (4.80 x 10^-4^) and FDR bottom line (4.09 x 10^-4^). All other behavioural variables are distinguished by plotting colour (Demographic = yellow, Health = red, Lifestyle = blue). Abbreviations: IQ-11, IQ-20, IQ-57, IQ-63 = general intelligence score at ages ~11, ~20, ~57, and ~63; MOT = motor task; ME = mean error; ML = mean latency; PAL = paired associates learning; TE adjusted = total errors adjusted; TT Adjusted = total trials adjusted; PRM = pattern recognition memory; SD = standard deviation; CL = correct latency; RTI = reaction time task; MT = movement time; RT = reaction time; RVP = rapid visual processing task; MLB1-4 = mean latency block 1 to 4; SOC = Stockings of Cambridge; Mean Initial TT 5 Moves = mean initial total time 5 moves task; Mean Subse TT 5 Moves = mean subsequent thinking time 5 moves task; SRM = spatial recognition memory; TM = trail making task).

All cross-sectional-longitudinal and longitudinal univariate correlations between brain IDPs and behavioural measures revealed no statistically significant relations when accounting for multiple testing (FDR > 0.05). Specifically, for longitudinal correlations, this was the case for correlations adjusted and unadjusted for the effect of average scores ((W-63+W-57)/2). We visualize results with Manhattan plots that show -log _10_ p-values for IDP-by-behavioural longitudinal correlations, arranged by behavioural measures on the x-axis, multiple testing thresholds across all pairwise associations are marked with a horizontal line, FWE top line (P_uncorrected_ = 6.01 x 10^-6^) and False Discovery Rate (FDR) bottom line (P_uncorrected_ = 5.03 x 10^-5^), Figure 1. Similarly, we visualize results with Manhattan plots that show -log_10_ p-values for cognitive-by-all-other-behavioural cross-sectional-longitudinal correlations, arranged by cognitive variables on the x-axis; multiple testing thresholds across all pairwise associations are marked with a horizontal line, FWE top line (P_uncorrected_ = 4.80 x 10^-4^) and FDR bottom line (P_uncorrected_ = 4.09 x 10^-4^), Figure 2.

### Bland-altman plots

In addition to univariate correlations, we use Bland-Altman (BA) plots to assess the relation between longitudinal change in normalized IQ score from childhood (age ~11), youth (age ~20), and late midlife (ages ~57 and ~63) at different magnitudes of the measured (mean) IQ score. Specifically, BA plots presented in Supplementary Figure 3 do not exhibit any particular structure, as (e.g.) might be expected if high IQ subjects had relative greater change. Rather, the BA plots indicate that the direction and magnitude of change in IQ is unrelated to mean cognitive ability in our sample.

### Multivariate associations

### Whole-group multivariate associations

CCA identified a single statistically significant mode of population co-variation coupling longitudinal cross-subject variations in brain structure to an extensive range of behavioural measures (R_c_ = 0.9, permuted P_corrected_ = 0.001). Post-hoc correlational analyses indicated that decreases in cognitive performance (IQ-57 – IQ-20) and speed assessed tasks, higher rates of familial myocardial infarct, lower HDL cholesterol, less physical activity, and higher scores on the mental depression inventory are associated with larger decreases (from age ~57 to ~63) in whole brain GM and WM volume but increases in some WM and GM ROIs, in particular the GM cerebellum. Finally, we did not find evidence supporting the role of baseline scores as predictors of impending brain or behavioural change in late-midlife

**Figure 3:**
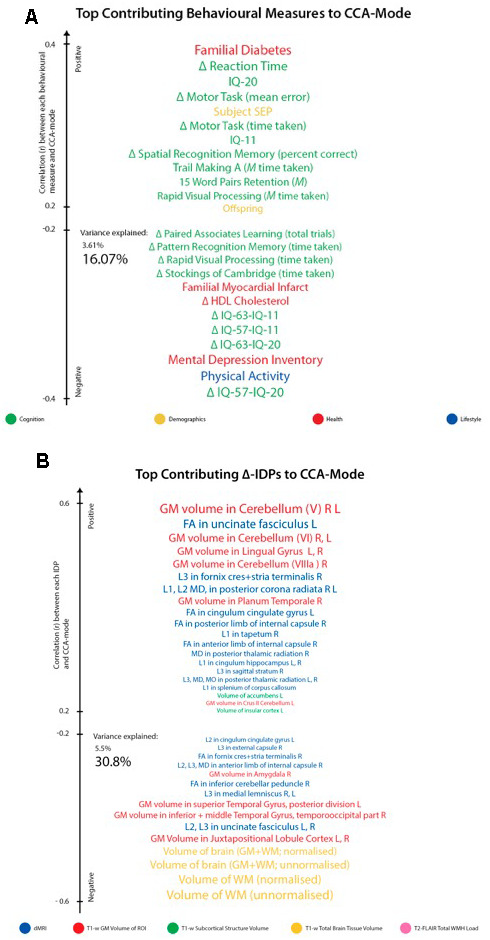
**Top contributing variables to identified CCA-Mode using post-hoc correlational analyses.** Individual behavioural (**A**) and IDP (**B**) measures most strongly associated with the CCA-mode of population covariation. The CCA-derived weights visualized indicate how much each measured variable contributes to the significant CCA-mode i.e., the measure of the strength of involvement of an observed variable to the CCA-mode, derived from post-hoc correlational analyses. Behavioural measures and IDPs are coloured according to their assigned subdomains. The vertical position of each variable is related to the scale of the association of that specific measure with the identified CCA-mode. Font size is indicative of variance explained by the CCA-mode. Here we do not report variables that attain a correlation value between 0.2 to -0.2. Abbreviations: Δ = longitudinal change (W-63 – W-57), M = average ((W-63 + W-57)/2), L = left, R = right, FA = fractional anisotropy, L1 = 1^st^ eigenvalue, L2 = 2^nd^ eigenvalue, L3, = 3^rd^ eigenvalue, MD = mean diffusivity, MO = tensor mode.

For ease of interpretation and comparison with an earlier study, we invert the signs of all baseline (W-57) and follow-up (W-63) behavioural measures where lower values reflect higher cognitive performance or more favourable/healthy traits (e.g., speed assessed tasks, number of total errors, total cholesterol, BMI). Thus, in general, using Figure 3A, we interpret positive post-hoc correlations between each observed behavioural measure and the CCA-derived subject weights (i.e. canonical variate weights, U or V) as positive or “healthy” contributions to the CCA-mode, whilst all negative behavioural correlations are interpreted as negative or “unhealthy” contributions. However, as MRI-derived brain structural measures are themselves indirect measures of underlying brain neuroanatomy, we cannot be entirely certain what an observed brain measure or difference (i.e. change) score truly represents. Considering this uncertainty, assigning a “good” or “bad” direction to estimated longitudinal change scores in a brain biomarker is extremely risky and avoided in this study.

Post-hoc correlational analyses identified the strongest positively associated behavioural variable to the CCA-mode as (self-reported) familial history of diabetes (*r^2^* = 10.5%, *r*=0.33) and the strongest negatively linked variable as longitudinal change in IQ (IQ-53 – IQ-20; *r^2^* = 16.1%, *r*=0.40), Figure 3A. Other strong positive behavioural contributions to the CCA-mode include change in motor and reaction time task (RTI), youth IQ (IQ-20 and IQ-11), change in spatial recognition memory task (SRM percent correct), average score in processing speed (Trail Making A), average score in verbal paired associative learning and memory (15 word pairs retention), subject SEP and offspring. Other strong negative behavioural contributions to the CCA-mode include: change in IQ variables (IQ-63 – IQ-20, IQ-57 – IQ-11, IQ-63 – IQ-11), change in speed assessed tasks executive functioning/planning (SOC), change in attention with working memory load (RVP) task, change in pattern recognition memory (PRM) task, change in paired associates learning (total trials), and a number of lifestyle and health variables (e.g. physical activity, MDI score, familial history of myocardial infarct, and HDL cholesterol).

With regards to post-hoc correlations computed between IDPs and the CCA-mode, Figure 3B, we identified longitudinal change in GM volume of the cerebellum (right) as the strongest positively linked brain-imaging measure (*r^2^* = 30.8%; *r* =0.56), and longitudinal change in total WM volume (normalized for head size) as the strongest negatively linked (*r^2^* = 35.0%; *r* =-0.59). Other top contributing positive IDPs include change in GM volume of cerebellum and non-cerebellum regions-of-interest (ROIs), change in the diffusion properties of several microstructural WM ROIs, and change in the volume of subcortical structures, the nucleus accumbens (Nac) and caudate. Post-hoc correlational analyses also identified a number of strong negatively contributing IDPs to the underling structure of the identified CCA-mode. Of these, the most influential include: change in global brain volume (grey and white matter), change in GM volume of cerebellum ROIs (the juxtapositional lobule cortex, inferior and superior temporal gyrus), and a range of dMRI microstructural markers (medial lemniscus, cerebellar peduncle, internal and external capsule, crus fornicis and stria terminalis, cingulum cingulate gyrus, uncinate fasciculus).

While no one result can be taken in isolation, we pull out just a few variables to illustrate the directions of the effects: Higher rates of familial myocardial infarct, less physical activity, higher score on the mental depression inventory (MDI), decreases in cognitive performance (IQ-63 - IQ-11, IQ-57 - IQ-11, IQ-63 - IQ-20), and speed assessed tasks is associated with larger decreases (from age ~57 to age ~63) in whole brain GM and WM but increases in some WM and GM ROIs, in particular in the GM cerebellum.

**Figure 4:**
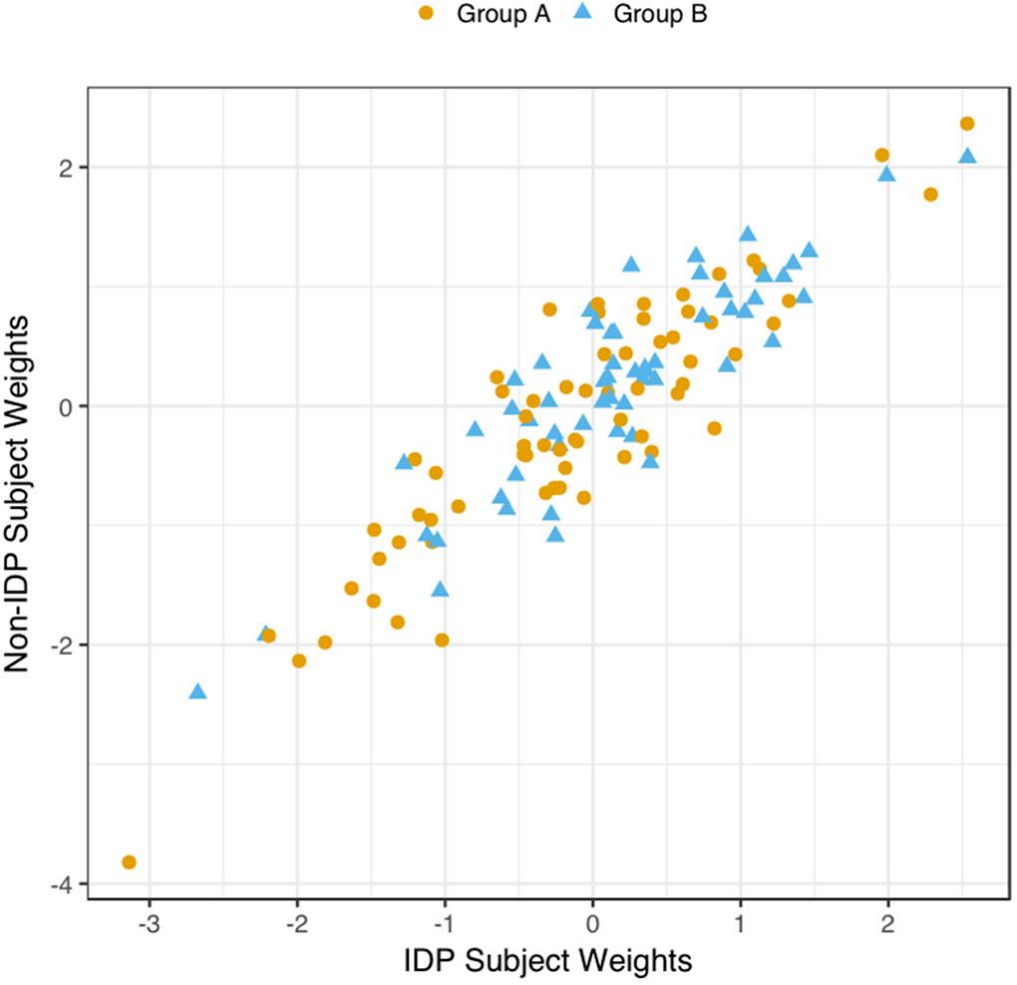
**Scatter Plot of all Subject’s Relationship with the Significant CCA-Mode.** The scatter plot shows the association between individual subject weights from the IDP canonical variate (V) versus individual subject weights from the behavioural canonical variate (U), with one point per subject. The high correlation observed here reflects the significant covariation between the behavioural and imaging longitudinal datasets. Group membership is indicated by plotting symbol (orange circle = group A i.e. improvers; blue triangle = group B i.e. decliners).

We explored the multivariate results to establish that the estimated CCA-mode was not unduly influenced by the EGD. Specifically, a scatterplot of the IDP and behavioural canonical variates, with group membership indicated by plotting symbol, showed no evidence of clustering, Figure 4.

## DISCUSSION

This study makes a number of key findings: First, CCA revealed a single significant mode of population covariation that linked multiple longitudinal measures of brain-imaging structural measures to multiple longitudinal behavioural measures (R_c_ = 0.92, permuted P_corrected_ = 0.001). Specially, this discovery indicates that variance is shared across longitudinal measures of cognition, demographic, health and lifestyle factors, and that this explains variance in important longitudinal brain structure measures. Second, post-hoc correlational analyses between the significant CCA-mode and the observed variables suggest that participants demonstrating cognitive decline (across specific cognitive domains) also show decreases in total brain volume within the two late-midlife assessment intervals. This finding lends support to the concept of a general intelligence, or g-factor – used to describe the interrelation among diverse mental abilities [47] – but extends it to include the contribution of brain ageing and other aspects of real-life function e.g. socioeconomic factors, mental health, lifestyle behaviours. Specifically, the discovery of this CCA-mode indicates two key points: First, the results corroborate the existence of a domain-general mechanism that is impaired by normal (non-pathological) ageing processes – which in this study is reflected by potential age-related biomarkers and environmental factors to the end effects (i.e. cognitive decline and brain atrophy). Second, the differential association of brain and behavioural longitudinal measures with chronological age indicate that different systems do not “all go together when it goes”, but rather that different aspects of behaviour and biology may be characterised by their own age-trajectory [48]. Our results did not find evidence supporting the role of baseline (W-57) or follow-up (W-63) cross-sectional measures as predictors of impending change in late-midlife. The latter is consistent with mixed findings in the literature that also found variables correlated cross-sectionally at baseline are not inherent predictors of subsequent change. Finally, controlling for the effect of average ((W-57+W-63)/2) scores on longitudinal correlations did not alter our results.

Consistent with prior studies [7, 12–14, 19], we found that age-related total brain volume loss was linked to decline in several cognitive domains. That is, our results indicate that participants who experienced a decrease in cognitive performance over the inception of the study, were also those less resistant to the various elements driving brain atrophy. Interestingly, the cognitive measures most strongly associated with the CCA-mode of population covariation (identified using post-hoc correlations) are consistent with the cognitive domains well-established for their increased vulnerability to advancing age [2, 7, 9, 35, 49, 50], Figure 3A. Specifically, post-hoc correlations between the significant CCA-mode and the observed behavioural variables coupled decline in cognitive measures assessing general intelligence (IQ), executive functioning (SOC), attention with working memory load (RVP), pattern recognition learning and memory (PRM), visual paired associates learning and memory (PAL) to poorer mental health (assessed by MDI), decreased physical activity, lower HDL-cholesterol, a familial history of myocardial infarct, higher body mass index (BMI), higher alcohol consumption, and smoking. Here, our results suggest a link between negative lifestyle behaviours and age-related decline and are thus consistent with prior age-related studies that have similarly identified associations linking higher general intelligence and bigger brain volume to greater physical fitness and (other) positive lifestyle behaviours in older adults [2, 14, 17, 18, 51–55]. Finally, we found that covariation in the aforementioned behavioural measures were important to declines in the following brain-imaging variables; total white matter (WM) volume, total brain volume (GM and WM), GM volume of the juxtapositional lobule cortex, temporal gyrus related regions-of-interest (ROIs), and a range of WM (microstructural) brain ROIs.

Notably in this study, we apply caution when interpreting the subject-SEP and physical fitness related findings. First, the indicators of subject-SEP were limited to one item (working or not working) and second, the significant role of physical fitness may, in part, reflect pre-existing genetic differences which renders moot any implication that improving physical fitness in late-midlife is causally related to one’s ageing trajectory. Future investigations that use more than one indicator of subject-SEP, and are able to examine the effects of pre-existing genetic differences in physical fitness can further elucidate the role of these measures in age-related trajectories. Nonetheless, irrespective of whether these relations underlie causality, the present findings suggest that variations in the level of physical fitness and subject-SEP may be partially accountable for the individual variability observed in normative ageing trajectories.

This study explores the contribution of multiple inter-related risk and protective factors to age-related (brain-behaviour) changes simultaneously. There are several advantages to this approach. First, studies including only a small number of potential age-related covariates risk identifying relations that may in part, or entirely, be artefacts of relations not accounted for. Thus, our ability to account for the effects of a wide-range of measures can potentially attenuate this type of confounding whilst also providing a more realistic setting for identifying potential contributors of age-related decline. Second, although contributors or correlates of age-related differences in brain structure and cognitive function may individually be of negligible consequence to later-life health outcome, it has been demonstrated that their cumulative effects may be of importance to the observed heterogeneity in ageing trajectories [14, 18, 56]. The advantage of modelling multiple heterogeneous variables simultaneously is demonstrated in randomized controlled studies investigating the effects of lifestyle behaviours on health outcome. In one example, the mutual interrelation across multiple diverse measures was shown through the increased success of intervention programs that targeted multiple health behaviours simultaneously or sequentially over programs that isolated single measures [57]. Specifically, “simultaneous multiple health behaviour research” promotes the benefits of multiple intervention targets compared to programs that value specificity above all else. It is suggested that the silo mentality of traditional scientific research greatly impedes our ability to recognize the commonality across diverse traits, and in general cautions researchers against the compartmentalization of anatomical structures, physiological processes and behaviours as unitary, unrelated constructs. Thus, with growing evidence supporting an integrative, multidisciplinary approach to health interventions, multidimensional studies like ours are found to be more favourable in yielding reliable and informative results.

**Figure 5:**
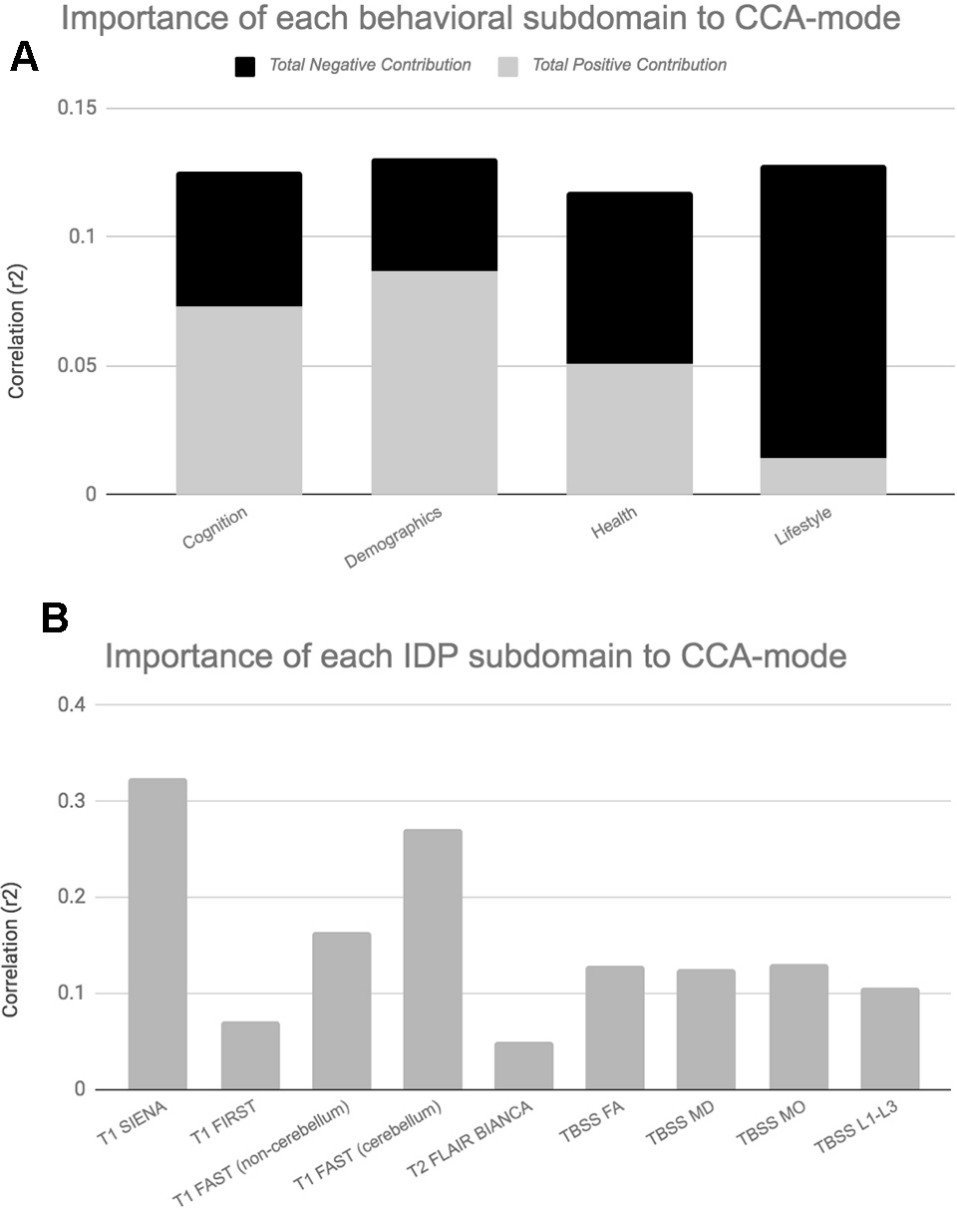
**Importance of Behavioural and Brain-Imaging Subdomains to the CCA-Mode.** Figures 5A, 5B visualize the overall significance of behavioural (**A**) and IDP subdomains (**B**) in influencing multivariate associations between each variable included in the measurement battery. For each subdomain (x-axis), the length of each bar represents the average subdomain importance (r^2^) to the CCA-mode. For behavioural subdomains only, categorically-driven contributions from positive qualities or indicators are represented in grey, whilst contributions from negative traits are represented in black. For brain-imaging subdomains all contributions are shown in grey as interpretation of the direction of longitudinal change observed in brain biomarkers was avoided due to the associated uncertainty. In this study, individual measures describing whole brain tissue volume (SIENAX and SIENA) and demographics were identified as the most important contributors to the CCA-mode of population covariation. Abbreviations: FA = fractional anisotropy, L1 = 1^st^ eigenvalue, L2 = 2^nd^ eigenvalue, L3, = 3^rd^ eigenvalue, MD = mean diffusivity, MO = tensor mode, FAST = FMRIB’s Automated Segmentation Tool, FLAIR = Fluid-Attenuated Inversion Recovery, BIANCA = Brain Intensity AbNormality Algorithm, TBSS: tract-based spatial statistics.

When evaluating the top contributing brain-imaging measures identified by post-hoc correlational analyses, our findings are in agreement with previous reports that also link changes in total brain volume (GM and WM) to changes in cognitive ability [7–9, 40, 46], Figures 3, 5. According to Stern’s reserve hypothesis, larger brains - which ostensibly reflect greater neuronal density and more extensive synaptic networks - are less vulnerable to the effects of ageing than smaller ones, therefore MRI-derived measures of total brain volume which presumably represents the volume of neuropil are considered to be top indicators of overall brain health and related cognitive function. However, there are several caveats to interpreting the neurobiological processes or environment inferred by MRI brain-imaging. First, as MRI can only offer an indirect measure of brain structure, it is unclear whether measured differences in brain volume across the two consecutive assessment intervals - both between and within subjects - genuinely reflect underlying age-related neurobiological processes or errors of measurement. Thus, with the uncertainty of what the estimated MRI-derived brain change truly reflects, further interpretation of how these measures relate to cognitive change will at best be an approximation. However, notwithstanding the ambiguity of the physical substrate being measured, it is unlikely that any one neurobiological process is accountable for the dynamic structure-function relations observed in normal ageing. Under the premise that the brain is the physical substrate of behaviour, subjects experiencing brain atrophy – i.e. indicating loss of neuropil and neural connections – are typically expected to demonstrate cognitive decline, a theory supported by the negatively contributing brain and behaviour measures to the CCA-mode, Figure 3A, 3B. Conversely, if an increase in brain volume is an indicator of pathological processes such as gliosis, inflammation, or defective elimination of by-products [9], we would expect to observe relations that link increases in brain volume to declines in cognitive ability. The CCA results are also consistent with this theory as we also identified highly contributing positive contributions from GM volumes of cerebellum ROIs (lingual gyrus, planum temporale, accumbens, insular cortex) and DTI-indices fractional anisotropy (FA), mean diffusivity (MD), axial diffusivity (AD) and radial diffusivity (RD) to declines in cognitive performance. A third scenario – consistent with both Stern’s active reserve hypothesis and the scaffolding theory of ageing and cognition (STAC) [58, 59], postulates that irrespective of the extent of age-related (pathological) brain change or the amount of initial brain reserve capacity (i.e. brain size, neural count) [59], the ageing process is kinder to individuals with higher baseline intelligence and those who engage in cognitively or socially stimulating activities. Thus, with these preconditions in mind, individual differences in cognitive ability and decline are proposed to be partly attributable to cognitive reserve proxy measures (e.g. childhood intelligence, SEP, extracurricular activities) that are thought to promote functional adaptation and reorganization of brain elements in spite of age-related pathological change to maximise performance. If this were correct, the post-hoc CCA results linking brain atrophy and loss of WM integrity to increasing cognitive scores would also be accounted for. In summary, the CCA results revealed ageing patterns that may underlie: 1. longitudinal correlations between brain atrophy and cognitive decline, 2. correlations between increased brain volume (underlying potential age-related neurodegenerative processes) and cognitive decline, and 3. preservation of cognitive ability despite age-related pathological brain change due to the positive contribution of cognitive reserve proxy measures (i.e. evidence of active reserve) to later-life cognitive performance. Figures 3B, 5B present the relative importance of individual and subdomain imaging-derived contributions in maximising the correlation between the brain and behaviour datasets respectively. Specifically, when considering the significance of each IDP subdomain to the CCA mode (Figure 5B), we found that although total brain tissue volumes, volumes of subcortical brain structures, total WMH load, and non-cerebellum ROIs were predominantly negatively contributing, cerebellum ROIs and DTI indices (FA, MD, AD, RD) showed mixed contributions. However, when assessing the top contributing DTI measures to the identified CCA-mode, we found that the large majority of these measures were also negatively contributing. The pattern of broadly negatively contributing IDPs to the CCA-mode may indicate linked neurobiological processes such as demyelination, axonal degradation or gliosis that are responsible for the age-related brain atrophy associated to later-life. Furthermore, given the well-established link between WM integrity and speed-assessed tasks, memory, and executive function, our results also provide evidence towards the notion that changes in WM microstructure (as indexed by dMRI), in concert with total brain volume atrophy, are perhaps partly accountable for the declines observed in specific age-sensitive cognitive domains [5, 60–63].

Contrary to prior studies, we found no evidence of cross-sectional measures of brain or behaviour as predictors of longitudinal change [11, 12, 17–20, 51, 64]. This finding suggests that specific patterns of initial baseline scores may not necessarily offer greater or lesser immunity in the face of forces that drive age-related decline. Specifically, Supplementary Figure 3 shows low agreement (but unstructured) Bland Altman plots that do not indicate a relationship between the rate, direction or magnitude of change in IQ and initial (mean) cognitive ability. This is also confirmed in Supplementary Figure 1. Here the plot of normalized trajectories of cognitive change indicates that pre-existing between-person differences are preserved into later life, an example of ‘preserved differentiation” i.e., brighter children become brighter adults, but that this does not necessarily infer protection against the rate or onset of cognitive decline in later-midlife. Our findings are consistent with several other studies that investigated whether “age is kinder to the initially more able” [18, 21, 65]. Notably, the pattern of longitudinal change observed in Supplementary Figure 1 also brings to the forefront our use of the extreme group design (EGD) for subject recruitment. The main purpose of using this approach was to ensure that the variability in cognitive decline across time and subjects was sufficient to detect biological correlations in what is otherwise a modestly sized sample of healthy, homogenous subjects.

Although this study identified relations between brain-behaviour measures that are in agreement with prior ageing studies, we also report a number of inconsistencies. Below we provide likely explanations for these variations. First, findings from earlier studies assessing the interdependence of age-related change is mainly limited to data acquired using a cross-sectional design [21, 37, 46]. In general, cross-sectional and longitudinal studies have shown low agreeability, namely with cross-sectional studies underestimating the rate of age-related decline [11, 20, 21, 37, 64]. Unlike longitudinal designs that can provide reliable estimates of within-subject differences, rates of change therein, and the associations between these changes, cross-sectional studies can inherently only provide information on population-level mean trends and are therefore poorly suited for investigating true longitudinal change. Furthermore, cross-sectional studies that include a wide age-range are highly vulnerable to cohort effects, secular trends, and confounding by other unmeasured differences that may have been brought into the study from previous years. Evidently, such factors make cross-sectional studies suboptimal for assessing the relation of brain-behavior changes over time and may be partly accountable for the discrepant findings across studies. Second, it may be that, at least in part, only extremes of a variables range are related to the magnitude of change, rate of change, or to other age-related variables. As such extreme scores in behavioural or health indicators are most often observed in older adults or patient groups, it is likely that our healthy late-midlife sample may not have accrued sufficient age-related changes to meet the effect sizes required for these associations. Third, the lack of statistically significant univariate associations in this study may be due to the choice of model itself. For example, in one study [18], the contribution of physical fitness - when assessed as three single measures - to cognitive ability was small. However, when the individual fitness measures were replaced with a latent factor reflecting ‘overall fitness’, large associations with cognitive decline were identified. This finding suggests that broad latent measures of behaviour or health status, rather than narrow single indicators, are necessary to identify significant associations between age-related processes. Furthermore, although in some cases bivariate models are an obvious choice (e.g., exploring relations between specific brain regions hypothesized to mediate specific pathological behavioural changes), in the event of identifying modifiers or correlates of healthy ageing, it is most likely that the cumulative effect of a range of health and behavioural factors are responsible for the observed heterogeneity among individuals and thus should not be assessed by simple pairwise correlations alone. Fourth, our sample size – although comparable to other midlife longitudinal ageing studies – may be under-powered to detect the small effect sizes of “healthy” brain-behavior relations, especially if the variation in the brain-behaviour changes are small to begin with. Fifth, the lack of significant univariate correlations compared to earlier studies may also be attributable to a lead-lag interval that is not agreeable with the timing of effects between measures. Specifically, it is unreasonable to expect an immediate contribution from early changes in the presumed “causal” variable and subsequent change in the presumed “effect” variable. In this scenario, future investigations that employ lead-lag analyses using more than two assessments are necessary to prevent this type of limitation. Lastly, the inconsistent findings across studies may also reflect the variability in subject characteristics (e.g. age, ethnicity, geographical) or heterogeneity in tests used to measure similar constructs. For example, a study reporting an association between baseline hippocampal volume as a contributor of subsequent decline in memory in subjects who are 80+ years have a far greater chance of detecting relations that may not yet have reached the necessary effect sizes in subjects who are almost two decades younger and of better general health.

### Strengths and limitations

A major strength of this study is the study-sample itself. As MDBC-1953 is a healthy, homogenous, single-year-of-birth male cohort, subjects are ethnically, culturally and geographically homogenous, and of general good health. This setup dramatically minimizes the unwanted effects of chronological age and the potentially troublesome contributions of disease, differential environmental factors, and population stratification to variations in health outcome. A further strength of this study pertains to its use of CCA to explore longitudinal associations [89]. Specifically, CCA boosts power by using the full dataset to extract latent factors that are based on the shared variance observed among sets of related measures. This approach allows the simultaneous prediction of multiple outcome variables, permits the isolation of distinct biological mechanisms, and largely attenuates the unexplained residual variance through its identification of multiple modes. Next, unlike many longitudinal studies that are biased by selective attrition (i.e. due to the “3M” – mobility, morbidity and mortality [46]), of the 193 subjects who attended W-57, 64% returned to provide follow-up data at W-63. Another strength of this study concerns the manner in which subjects were selected. That is, compared to conventional ageing studies that are biased towards higher educated, more intelligent subjects, we recruited participants on the basis of cognitive change from youth to late-midlife based on the Extreme Group Design (EGD) [66]. Specifically, by sampling subjects from the extremes of the change-in-IQ distribution, our approach maximizes the variability in participant characteristics (e.g. cognitive ability, occupational complexity, levels of motivation) and with it the applicability of our findings to the general population. Equally important, is the ability of EGD to maximise the variability in cognitive decline in order to detect biological correlations. Further strengths of this study concerns the age-range of participants. Specifically, there is a lack of evidence indicating that pathological change begins abruptly at old age. Instead, a growing body of research has converged in highlighting the importance of early-life and midlife measures in predicting later-life health outcome [31, 42, 58, 67–70]. In view of this, the available early-life data, in combination with the late-midlife measures allows us to assess the contribution of potential modifiers and the interdependence of age-related processes prior to confounding by overt or underlying later-life pathological conditions. Lastly, our study includes a comprehensive range of broad brush and specific cognitive tests, brain biomarkers, and age-related covariates reducing the amount confounding that is attributable to associations driven by other unmeasured factors. Furthermore, “specific” measures (e.g. cognitive tests measuring a particular skill, or a brain ROI) are hypothesized to form stronger links than general “broad brush” measures (e.g. total brain volume, general intelligence).

This study also reports a number of limitations. First, we acknowledge that a major limitation of the present study lies in its modest sample size, decreasing the studies power to detect modest-to-small effects. Second, although repeated measurements are what allow longitudinal studies to assess change over time, the use of identical tests at each assessment increases the risk of underestimating age-related changes. This is mainly attributable to repeat exposure to the testing material, environment, and operator which ultimately result in practice-related learning. Thus, it is possible that accrued familiarity to testing conditions and materials may explain the observed gains in some of the variables examined. Notably, however, practice-related learning effects are only applicable to the cognitive tests administered in the two late-midlife waves. Relatedly, practice effects may also be partly accountable for the lack of statistically significant cross-sectional-longitudinal correlations. That is, the practice-related gains may have attenuated what was already only modest declines in cognitive ability in subjects that are a generally healthy and with it eliminating the opportunity of observing potentially important associations. That being said, prior studies formally investigating the contribution of retest effects to observed gains have reported on average moderate-to-large retest effects, but small inter-individual variability [71]. Specifically, this indicates that associations between ageing-related changes and their correlates identify true covariates of age-related cognitive change, rather than covariates of practice-related gains. The number of measurement occasions available for investigation may also be a limiting factor in this study. Specifically, as our study consists of two occasions of longitudinal testing we were unable to account for nonlinear trajectories of brain and cognitive changes. Lastly, the homogeneity of subjects also means that the findings reported in this study should be extending to the general population with caution.

## CONCLUSIONS

Our study demonstrates the benefits of using a homogenous, single-year-of birth cohort to examine the association between broad-brush and specific longitudinal measures of brain and cognition, and their relation to demographic, health and lifestyle factors. Here, we report correspondence between structural and functional changes that largely link brain atrophy to cognitive decline and negative self-care behaviours. However, we found no evidence in support of baseline or follow-up measures as predictors of impending brain or cognitive change, or that early intelligence level is protective against ageing-related cognitive decline. Instead, we confirm previous findings that identified total brain volume as a particularly informative indicator of underlying cognitive ability. Additionally, this study reveals several potentially influential modifiers of age-related trajectories: fitness level, mental well-being, subject-SEP, offspring, familial history of cardiovascular disease and diabetes, HDL-cholesterol, alcohol consumption, and smoking. Here, our findings endorse the notion that variability in life-course factors may play a key role in the rate, direction, and magnitude of age-related brain-behaviour changes and warrants further investigation using larger, more diverse samples, with more than two measurement occasions. As our study-sample are optimally healthy, the findings reported here provide evidence for associations that are relevant to healthy ageing and contribute to a better understanding of the brain as the physical substrate of cognitive ability in late-midlife.

## MATERIALS AND METHODS

### Participants: extreme group design (n=1,985)

The participants for this imaging sub-study were members of the longitudinal Danish Metropolitan Birth Cohort 1953 (MDBC-1953) [72]. For a detailed discussion regarding the subject selection criteria, recruitment, attrition and testing for this cohort see [72–74]. In summary, using youth and late midlife IQ scores, subjects were selected based on their estimated change in mental ability as part of an “extreme group design” (EGD) [75]. Specifically, the two well-validated tests, the Børge Priens Test (BP) [76] and Intelligenz-Struktur-Test 2000 R (IST) [77] were taken at ages ~20 (IQ-20) and ~57 (IQ-57) respectively. Since the cognitive change between these time-points was based on two different instruments, a change score was derived with a linear regression analysis of IQ-57 (IST-2000 R) on IQ-20 (BP) using a total of 1,985 subjects [74]. Both examinations comprise subtests that assess aspects of verbal intelligence (e.g. numerical series and verbal analogies), and thus are similarly structured and comparable. IQ-20 explained R^2^=50.4% of variance in IQ-57 (beta=0.71, p<0.0001), and we used each subject’s standardized residual about the regression line as a measure of their change in IQ across time. To avoid the effects of extreme test scores, subjects with absolute standardized residuals exceeding ±3 were omitted and the remaining members were classified into two groups pertaining to the degree of cognitive change observed from early-adulthood: group A = 66 improvers and group B = 57 decliners. This study has also been registered at clinicaltrials.gov (NCT03290040).

### Participants: present study (n=123)

**Figure 6:**
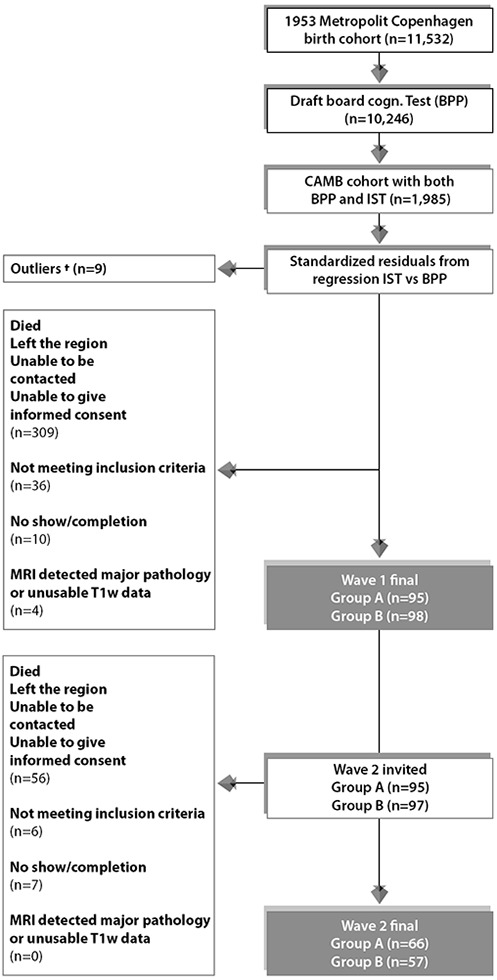
**Subject selection process for Wave 1/W-57 and Wave 2/W-63.** To avoid effects of extreme test scores, subjects with standardized residuals ±3 were omitted defined as here as †. The final sample size for the current study includes n=123 subjects who completed wave 1 and wave 2. Group A = 66 improvers and Group B = 57 decliners.

The majority of MDBC-1953 members have taken part in two early-life intelligence quotient (IQ) tests at ages ~11 (IQ-11) and ~20 (IQ-20). During this early-life period, information on early-life social and biological demographic factors was also acquired. Subsequently, in late-midlife, based on the degree of cognitive change, cohort members were selected (as described in section 1.1) to complete 2 waves of brain-imaging and behavioural assessments. The first wave (**W-57**, where 57 represents mean subject age at scanning, 57 years ±0.7 standard deviations (SD), took place during 2010-2013 and included a total of 193 subjects who had usable imaging and behavioural data. Data pertaining to W-57 have been previously investigated and reported [73, 78, 79]. The second wave, **W-63** (mean subject age 62.5±0.9 years) began in 2015 and is scheduled for completion in 2020. To date, of the initial 193 subjects with brain-imaging and behavioural data from W-57, 192 subjects were invited back to participate in W-63. 136 of the initial W-57 subjects accepted their invitation and proceeded to the subject screening stage. Here, participant eligibility was determined and this resulted in the exclusion of 6 subjects’ due to conditions related to substance abuse comorbid with cognitive impairment, psychiatric or neurological disease, and contraindications to MRI. Of the subjects who fulfilled the eligibility criteria, a further 7 were excluded due to no show on the day of examination or non-completion of MRI session. The final number of subjects in each group was: group A n=66, group B n=57. The average observation interval between W-57 and W-63 was 4.82±0.9 years. Figure 6 shows a recruitment flow diagram for the present study sample used to investigate brain and behaviour longitudinal correlations. Although there was a small range of ages during data acquisition, in general we refer to the ages of participants as 11 (W-11), 20 (W-20), 57 (W-57), and 63 (W-63) years. This imaging study was approved by the local ethical committee (De Videnskabsetiske Komiteer for Region Hovedstaden) and registered by the Danish Data Protection Agency. All participants provided written informed consent.

### Data

### Neuropsychological assessment: Repeated measurements

**Figure 7:**
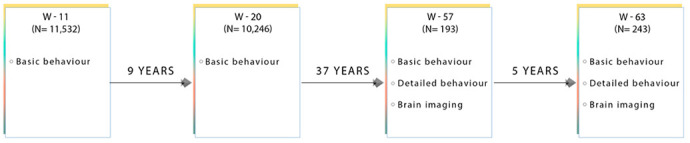
**Flow diagram showing the data collected at each time point for subjects used in this present sub-study.** W-11, W-20, W-57, and W-63 = data acquired at ages ~11, ~20, ~57 and ~63 respectively.

We include a detailed series of neuropsychological tests that were measured at both W-57 and W-63, Figure 7. All behavioural tests were acquired on the same day as the brain-MRI acquisition. In brief, global cognitive function was assessed with the mini-mental state examination (MMSE) and Addenbrooke’s cognitive examination (ACE). The Cambridge Neuropsychological Test Automated Battery (CANTAB) was administered to evaluate cognitive ability across the following cognitive domains: learning and memory (spatial and pattern recognition, and paired associates learning), executive function (planning), attention and reaction time [80]. Furthermore, we include both early-life measures of general cognitive ability acquired at W-11 (IQ-11) and W-20 (IQ-20) [81], as well as late-midlife measures of IQ acquired at W-57 (IQ-57) and W-63 (IQ-63). Table 1 presents the study sample characteristics for each cognitive examination used in the present analyses, separately for W-57 and W-63. To explore cross-sectional-longitudinal associations and longitudinal associations pertaining to brain and behaviour changes, we use cognitive scores acquired during W-57 and W-63 to estimate difference (i.e. change) (W-63 – W-57) and average ((W-63 + W-57)/2) scores. While it may seem more intuitive to compare change to a baseline, note that raw change is negatively correlated with baseline by construction. Thus, in the present analyses, each cognitive measure – with repeated measurements – is represented by its raw change and raw average counterparts and included as two independent measures of cognitive ability. Thus, in total we analyse 64 measures of cognitive performance of which 33 describe raw change, 27 describe raw average, and 4 describe IQ scores derived from W-11, W-20, W-57 and W-63.

### Demographic, health and lifestyle assessment:

We evaluate the effect of a range of reputed positive and negative measures on brain structure and cognitive performance. Assessments include both self-reported and objective measures such as, the Major Depression Inventory (MDI) [82], the Pittsburgh Sleep Quality Index (PSQI) [83], the Multidimensional Fatigue Inventory (MFI-20) [84], demographic factors, general health measures, history of non-communicable diseases (NCDs) and multiple health-related lifestyle behaviours. The majority of the potential age-related modifiers included were measured at W-63. Similar to neuropsychological tests with repeated measurements, demographic, health and lifestyle factors assessed more than once are included in subsequent analyses as raw change and raw average scores. These include: body mass index (BMI), total cholesterol (TC), high-density lipoprotein (HDL) cholesterol, low-density lipoprotein (LDL) cholesterol, and very low-density lipoprotein (VLDL) cholesterol. In summary, we report 8 = demographic, 34 = health, and 8 = lifestyle measures (total = 50), which together with the neuropsychological data is referred to as ‘behavioural’ measures. Finally, to assist the interpretation of results, behavioural measures were divided into four subdomains: cognitive, demographic (social and biological), health and lifestyle. See Tables 1–4 for a list of these variables and the study sample characteristics.

### MRI data acquisition

All subjects underwent whole-brain MRI scanning using a 3.0 T Philips Intera Achieva (Philips Medical Systems, Best, the Netherlands), with a 32-channel phased-array head coil. In all participants the following images were acquired during resting conditions: 1. anatomical high-resolution 3D T1-weighted (T1w) images using a gradient echo sequence (TR/TE = 6.9/700 ms; flip angle = 9°; voxel size = 1.1 × 1.1 × 1.1 mm^3^), 2. T2-weighted (T2w) (TR/TE = 1300/12 ms; flip angle = 90°, voxel size 1.8 × 1.8 × 9.5 mm), 3. T2w FLAIR (T2w-FLAIR) images (Fluid Attenuated Inversion Recovery) using turbo spin echo sequence (TR/TE = 11000/125 ms; flip angle = 90^0^, 6 slices, voxel size = 0.45 × 0.45 × 4.5 mm), and 4. diffusion weighted images (dMRI) (TR/TE = 9729/55; matrix =112 × 110 × 60; voxel size = 2 × 2.04 × 2 mm^3^) utilizing a single spin-echo echo-planar imaging sequence. For each dMRI scan, 33 images were acquired: 1 image with no diffusion sensitization (b=0 image), and 32 diffusion-weighted images (b = 1000s/mm^2^). Finally, all images were visually inspected in their raw state for bias field corruption, excessive motion, and other potential artifacts.

### Image analysis pipeline

We used the UKB image processing pipeline on our raw (non-processed) W-57 and W-63 brain-imaging data to enable future meta-analyses and replication studies [85]. Essentially, T1w structural data is used as the reference image to calculate cross-subject and cross-modality alignments required to process all other brain modalities.

In brief, we extracted 454 brain-imaging biomarkers (i.e., a broad set of biologically meaningful measures derived from multiple imaging modalities) that best capture the differential ageing processes and neuropathologies observed in a healthy ageing population. Subsequently, we categorized the extracted summary measures into 6 groups to reflect the MRI modality and image processing tool applied to derive each measure. These include: 1. T1w-SIENA percentage brain volume change (PBVC), 2. T1w-SIENAX (estimation of brain tissue volumes), 3. T1w-FIRST (segmentation of subcortical brain structures), 4. T1w total volume of grey matter (GM) in cerebellum and non-cerebellum regions-of-interest (ROI) [86, 87]^1^ using FAST-derived GM partial volume estimates (PVE), 5. T2w-FLAIR-BIANCA (total white-matter hyperintensity volume), and 6. dMRI-TBSS (microstructural properties of specific white matter (WM) tracts). With regards to the volume of GM in cerebellum and non-cerebellum ROIs, we inverted the non-linear registration to standard space which was subsequently used to warp a cortical atlas of 139 ROIs into native T1 space. Next, within each ROI we then summed the total volume of GM using the FAST-derived GM PVE.

Tables 5, 6 list all image-derived phenotypes measured in this study (also referred to as IDPs), their function and study sample characteristics.

**Table 6:** List of imaging-derived phenotypes (IDPs) obtained using the UKB image-processing pipeline.

**MRI Modality**	**Processing tool**	**Function**	**Description**	**No. of imaging variabes**	
				**W-57**	**W-63**
**T1-w**	FAST (FMRIB’s Automated Segmentation Tool) *	Segmentation of CSF, GM, and WM	Total volume of GM of cerebellum and non-cerebellum ROIs using GM partial volume estimates from FAST*	**139**	**139**
	FIRST (FMRIB’s Integrated Registration and Segmentation Tool)	Subcortical GM structure segmentation	Lateralized brain structures + brain stem	**15**	**15**
	SIENAX (single time-point)	Estimates brain tissue volumes (cross-sectional)	Global brain tissue volume (normalized and un-normalized for head size)	**10**	**10**
	SIENA (two time-points)	Detection of global GM atrophy (longitudinal)	Calculates percentage brain volume change (PBVC)	**-**	**1**
**T2-w (FLAIR)**	BIANCA (Brain Intensity Abnormality Classification Algorithm)	Quantification of total WMH volume	Total WMH volume	**1**	**1**
**Diffusion**	TBSS (Tract-Based Spatial Statistics)	Diffusivity estimates within 48 major WM tracts	Local diffusion properties reflecting integrity of microstructural WM tissue	**288**	**288**
				**TOTAL IDPS = 454**	

List of imaging modalities and processing tools employed (column 1 and 2), followed by the corresponding function of each processing tool (column 3), description of the brain phenotype extracted (column 4), and total number of IDPs generated for each brain-imaging category at W-57 and W-63 (columns 5 and 6). (FLAIR = fluid attenuated inversion recovery, CSF = cerebrospinal fluid, GM = grey matter, WM = white matter, WMH = white matter hyperintensity volume, ROIs = regions-of-interest). Total number of IDP measures n=454. *In order to obtain the GM volume of the 139 ROIs, we inverted the nonlinear registration to standard space, and used this to warp a cortical atlas of 139 ROIs into native T1 space. Within each ROI we then summed the total GM using the FAST-derived GM partial volume estimates. The 139 ROIs extracted are defined by a combination of parcellations from Harvard Oxford cortical and subcortical atlases, and Diedrichsen cerebellar atlas.

### Statistical analysis

We used univariate correlations and multi-level latent variable modelling to investigate specific and general relations between multiple brain and behaviour measures. To extract estimates of longitudinal change between two successive measurement occasions, we computed “raw difference scores” (RDS) (i.e., cross-sectional follow-up score at W-63 subtracted from cross-sectional baseline score at W-57). Several reasons motivated our selection of this approach. First, application of the commonly used “residualized change model” (RCM) can lead to biased results if the study sample consists of pre-existing groups at baseline [88]. Conversely, the RDS approach has shown to arrive at the correct inference regardless of whether there are pretest (baseline) differences in action e.g., when the association of a covariate or confounding variable with baseline scores is not equal to zero [88]. Second, although the latent difference score (LDS) model is not vulnerable to the aforementioned biases and may have been a natural choice to investigate longitudinal relations, its undeniable value is most apparent when constituent scores are derived using a well-defined instrument. However, as we include a wide range of variables that were acquired using a mixture of measurement methods from multiple measurement occasions the application of the LDS model was on this occasion unsuitable.

To investigate 1) the relation between cross-sectional baseline (BL_W-57_) and cross-sectional follow-up (FL_W-63_) brain and behaviour scores with longitudinal change (i.e., Δ_W-63-W-57_) referred to as “cross-sectional-longitudinal correlations”, and 2) coupled change between longitudinal brain and behavioural measures (i.e., Δ_W-63-W-57_ and/or ((W-63 + W-57)/2)) adjusted and unadjusted for the effects of average scores ((W-63 + W-57)/2), referred to as “longitudinal correlations”, we used both univariate (Pearson correlations) and multivariate statistics (canonical correlation analysis - CCA) [89], both analyses adjusted for a number of common confound variables, head size, motion during MRI, and age.

### Whole-group adjusted univariate associations

**Figure 8:**
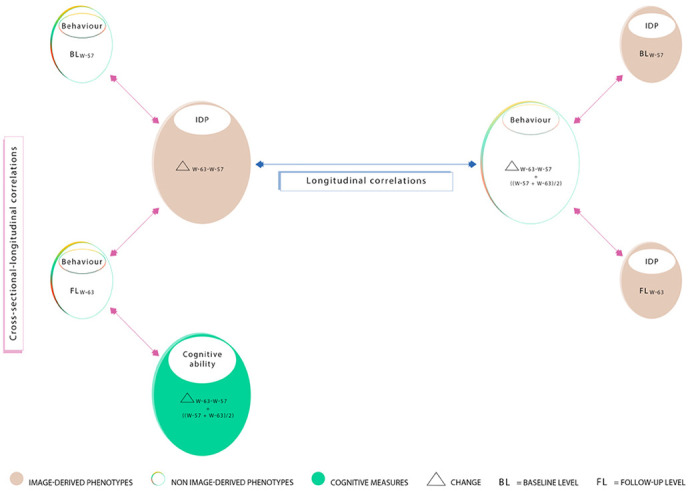
**Summary of correlations computed between measures acquired at W-57 (baseline; BLW-57), W-63 (follow-up, FL_W-63_), their differences i.e. change (Δ_W-63-W-57_), and average ((Δ_W-57+W-63_)/2).** Measured brain-imaging and behavioural variables from W-57 and W-63 are represented by smaller-sized ovals (1a-1d). Measures of change estimated using the difference between W-63 and W-57 and their averages are represented by larger-sized ovals (2a-2c). Ovals 1a and 1b represent 70 cross-sectional behavioural items and ovals 1c and 1d represent 453 cross-sectional IDPs. Ovals consisting of longitudinal variables (i.e. change and/or average scores) include 454 IDPs (2a), 114 behavioural items (2b), and 60 cognitive items (2c). In this study, we explore “cross-sectional longitudinal correlations” (pink arrows), and “longitudinal correlations” (blue arrow) using univariate and multivariate analyses. All correlations were adjusted for nuisance confounders motion (during MRI), age, and head size.

We used Pearson’s correlations to examine both cross-sectional-longitudinal correlations and longitudinal correlations (defined above) between each of the 454 (longitudinal) or 453 (cross-sectional) brain IDPs to each of the 114 (longitudinal and average) or 70 (cross-sectional) behavioural variables extracted from the MDBC-1953 database (full set of IDP x behavioural estimates: 1) 454 × 70 or 453 × 114 for cross-sectional-longitudinal correlations and 2) 454 × 114 for longitudinal correlations. Figure 8 provides a visualisation of all correlations computed between IDP and behavioural datasets for both univariate and multivariate analyses. Here, pink arrows indicate cross-sectional-longitudinal correlations and the blue arrow shows longitudinal correlations. Lastly, we also examined the relation between each of the 60 cognitive (longitudinal and average) measures to each of the (other) 50 (cross-sectional) behavioural variables (full set of cognitive variables × all (other) behavioural estimates: 64 × 50). In total, three variants of Manhattan plots are created to display the significance (-log10 P-values) of Pearson’s correlations for IDPs × behavioural estimates (1. cross-sectional-longitudinal correlations: 31,780 or 51,642 values; 2. longitudinal correlations: 51,756 values; and 3. cognition x all (other) behavioural measures (cross-sectional-longitudinal relations: 3200 values).

To reduce the influence of potential outliers and increase the reliability of associations made, we applied rank-based inverse Gaussian transformation (quantile normalization) to enforce Gaussianity for each of the brain IDPs, behavioural, and confound variables. For univariate correlations, missing data was handled with a complete case approach individually for each pair of variables; for CCA, where missing data is particularly problematic, we then applied an iterative PCA algorithm (based on the soft shrinkage of eigenvalues) to impute missing data values until convergence [90]. Finally, five confound variables were created relating to effects that may trouble the interpretation of computed correlations: absolute motion during MRI, relative motion during MRI, head size, age (difference (W-63-W-57) and average ((W-63+W-57)/2). The confound variables were regressed out of all brain-imaging and behavioural variables prior to correlational analyses. To account for multiplicity, we assessed the strength of significance with two types of multiple testing correction controlling the familywise error rate (FWE) via Bonferroni and the false discovery rate (FDR) [91].

### Extreme-group design validation test: Univariate associations

In order to assess the impact of the EGD, we separately compute univariate associations between each IDP and behavioural measure for group A (“improvers”) and group B (“decliners”) subjects. Each sub-analysis should be free of any spurious associations driven by average group differences in cognitive level (i.e., an example of Simpson’s paradox [43], whereby suboptimal pooling across variables such as cognitive level can potentially generate misleading associations).

### Whole-group adjusted multivariate associations

To explore the relation between multiple brain IDPs and behavioural measures simultaneously we applied CCA.

For the CCA, we adopted a similar approach as described previously [79, 85, 92]. In short, CCA was computed (canoncorr; MATLAB 2014a) following the model: U = AX and V = BY; where X represents the set of IDPs, Y is the set of behavioural measures, and A and B are optimized to maximize the correlation between each canonical variate pair, U and V [89]. The magnitude of the relationship between each variate pair is reflected by the canonical correlation coefficient (R_c_), an indicator of how strongly the estimate of population covariation is reflected in both IDP and behavioural datasets. Intuitively, we can think of CCA as identifying two latent variables, U_i_ and V_i_ (i.e. canonical variates whose elements we refer to as individual subject weights)_,_ from a specific linear combination of weighted MRI-derived brain measures that are most strongly associated to a specific linear combination of weighted behavioural measures.

IDP and behavioural datasets for CCA analysis were prepared using the same procedure as for the univariate correlation analysis. This resulted in a brain-IDP matrix of size 123 x 454 (subjects × IDPs) and a behavioural matrix of size 123 × 114 (subjects x behavioural measures) when investigating longitudinal correlations, and a brain-IDP matrix of size 123 x 453 (subjects × IDPs) and a behavioural matrix of size 123 × 70 (subjects x behavioural measures) when investigating cross-sectional-longitudinal relations. Typically, these datasets are the inputs fed into the CCA algorithm. However, to reduce overfitting (i.e., tending towards a rank-deficient CCA solution), prior to CCA we separately reduced the dimensionality of each dataset using PCA. Specifically, after accounting for missing data as before, we compressed the size of each matrix along the respective phenotype dimension to the top 30 subject-eigenvectors which accounted for ~70% of the total variance in our datasets (66.9% for IDPs, 76.1% for behavioural measures). The final dimension of each matrix fed into CCA was therefore 123 x 30 (subjects x PCA-derived components), with an output of 30 CCA modes estimated.

Statistical significance of the modes estimated was determined using 10,000 permutations of rows of one matrix relative to another. CCA was then re-run after each permutation and the respective r-values for each permuted CCA mode was estimated. Each observed canonical correlation r is compared to the null permutation distribution of the largest canonical correlation creating familywise error p-values corrected for searching over all 30 canonical correlation dimensions.

### Post-hoc correlations

To relate the CCA modes estimated back to the observed IDP and behavioural variables, we perform post-hoc correlations between each observed (quantile-normalised and deconfounded) variable with the canonical variate weights (U or V). This is analogous to the computation of factor loadings, and in CCA are formally referred to as canonical structure correlations. Generally, variables with larger post-hoc correlations indicate greater association with a CCA mode.

## Supplementary Material

Supplementary Figures

## Footnotes

1 The 139 ROIs were defined by a combining parcellations from the Harvard Oxford cortical and subcortical atlases, and Diedrichsen cerebellar atlas.
