# Supplementary material for: Discovering correlates of age-related decline in a healthy late-midlife male birth cohort
Source: Aging (Albany NY). 2020 Sep 10;12(17):16709–43. doi: 10.18632/aging.103345 (PMC7521526; doi:10.18632/aging.103345)
Supplement: Supplementary Figures [file aging-12-103345-s001..pdf]

SUPPLEMENTARY FIGURES

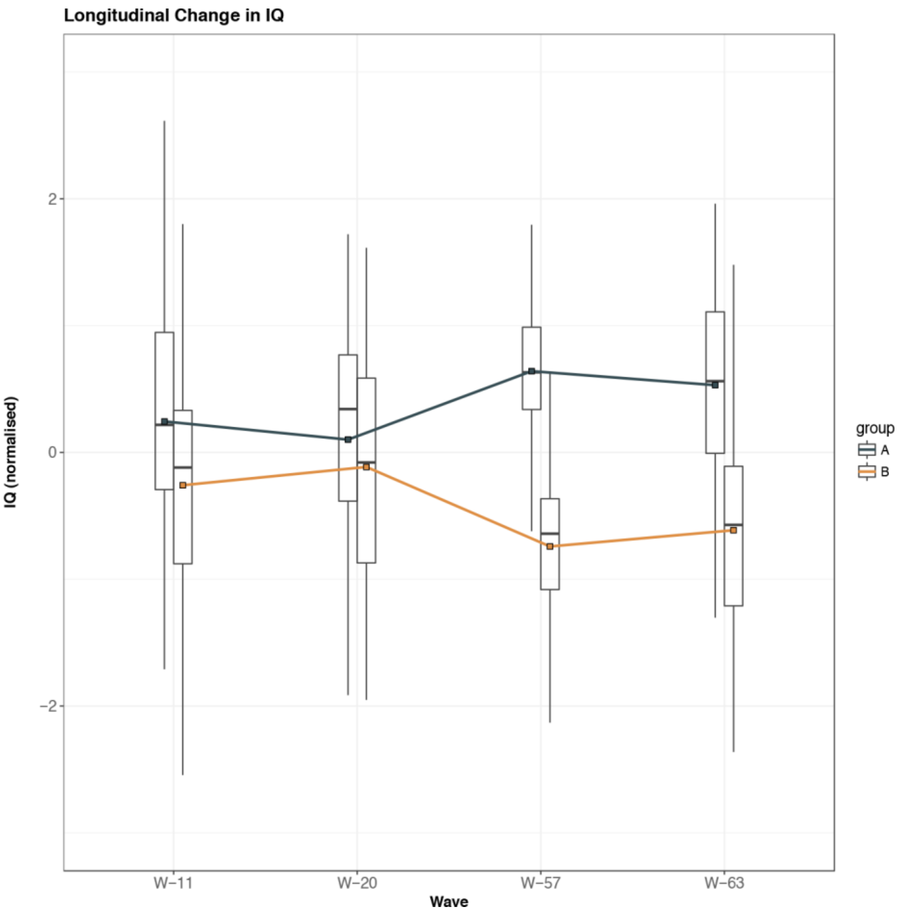

Supplementary Figure 1. Box-plot analysis showing group differences in the distribution of (normalized) intelligence quotient (IQ) scores from ages 11yrs (W-11) to 63yrs (W-63), with group membership identified by colour. IQ scores from time points W-20 (Wave-20) and W-57 (Wave-57) were used to define group members as “improvers” or “decliners” as part of the extreme group design i.e. using each subject’s estimated change in IQ from W-20 when subjects were ~20yr of age (BP test) and W-57 when subjects were ~57 (IST test). Abbreviations: W-11 = IQ score at mean subject age ~11 years, W-20 = IQ score at mean subject age ~20 years, W-57 = IQ score at mean subject age ~57 years, W-63 = IQ score at mean subject age ~63 years, Group A = improvers (grey), Group B = decliners (orange) as identified by the Extreme Group Design.

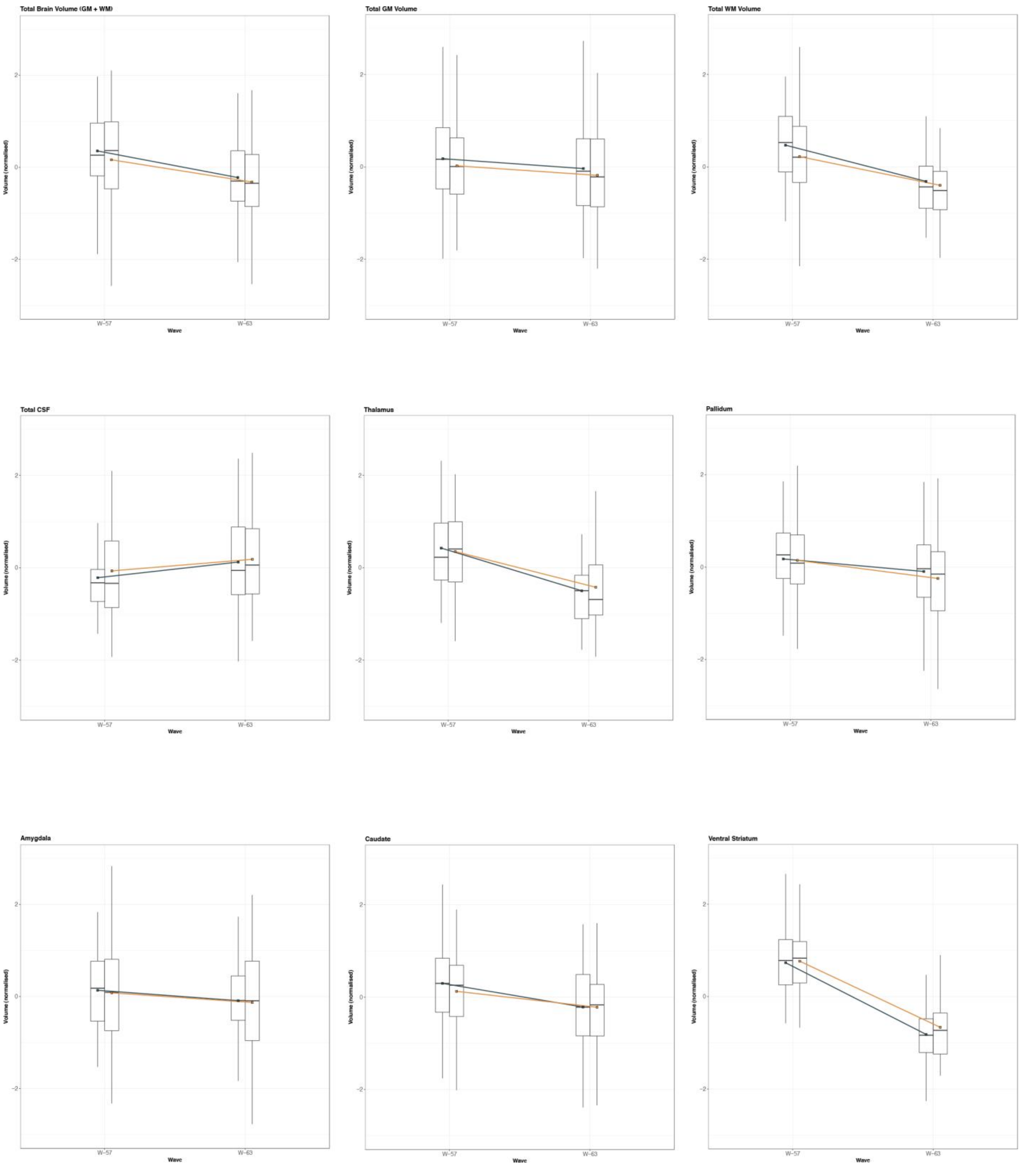

Total Brain Volume (GM + WM)
Total GM Volume
Total WM Volume
Total CSF
Thalamus
Pallidum
Amygdala
Caudate
Ventral Striatum
Volume (normalised)
Wave
W-57
W-63
2
0
-2

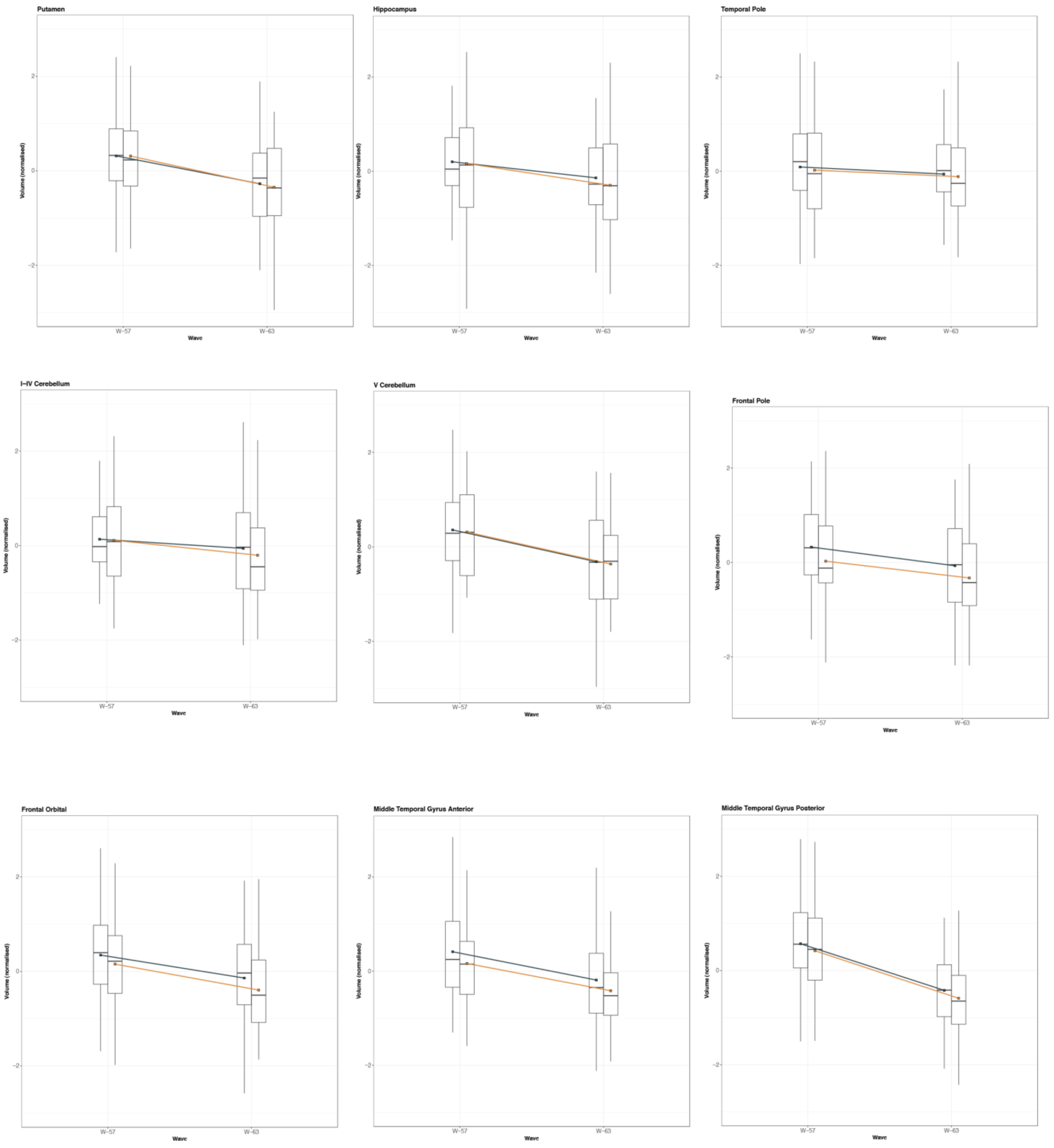
Putamen
Hippocampus
Temporal Pole
I–IV Cerebellum
V Cerebellum
Frontal Pole
Frontal Orbital
Middle Temporal Gyrus Anterior
Middle Temporal Gyrus Posterior
Volume (normalised)
Wave
W–57
W–63
2
0
–2

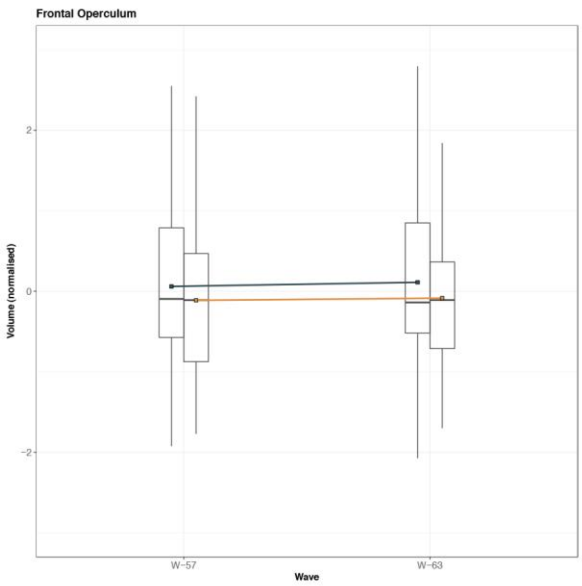
Frontal Operculum
Volume (normalised)
2
0
−2
W−57
W−63
Wave

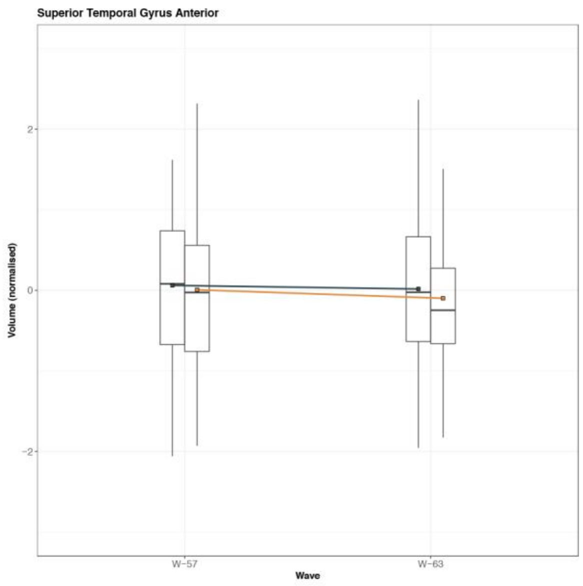
Superior Temporal Gyrus Anterior
Volume (normalised)
2
0
−2
W−57
W−63
Wave

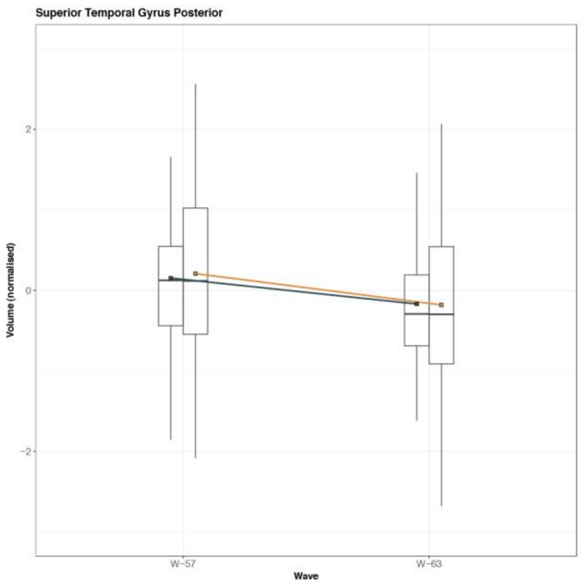
Superior Temporal Gyrus Posterior
Volume (normalised)
2
0
−2
W−57
W−63
Wave

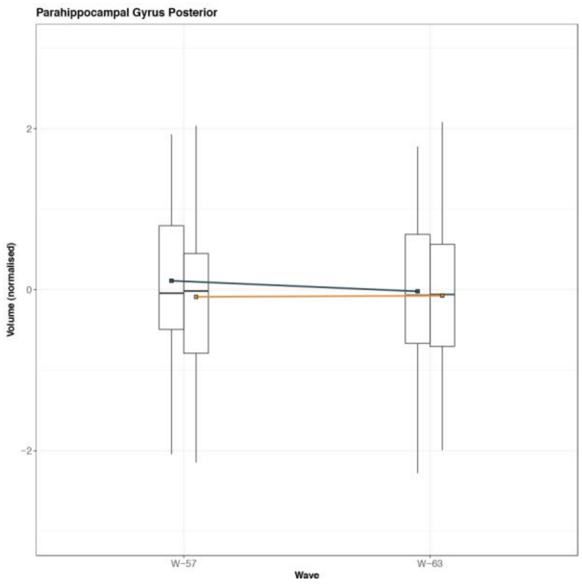
Parahippocampal Gyrus Posterior
Volume (normalised)
2
0
−2
W−57
W−63
Wave

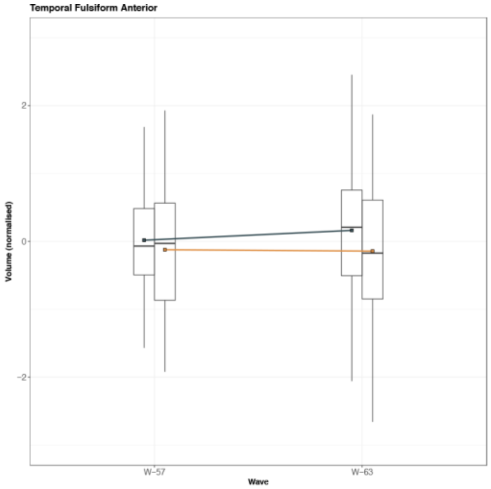
Temporal Fulsiform Anterior
Volume (normalised)
2
0
−2
W−57
W−63
Wave

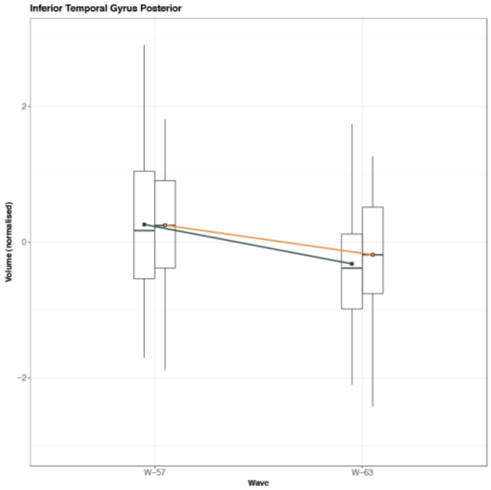
Inferior Temporal Gyrus Posterior
Volume (normalised)
2
0
−2
W−57
W−63
Wave

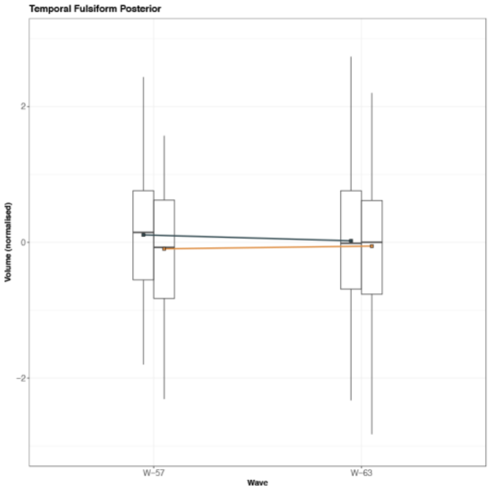
Temporal Fulsiform Posterior
Volume (normalised)
2
0
−2
W−57
W−63
Wave

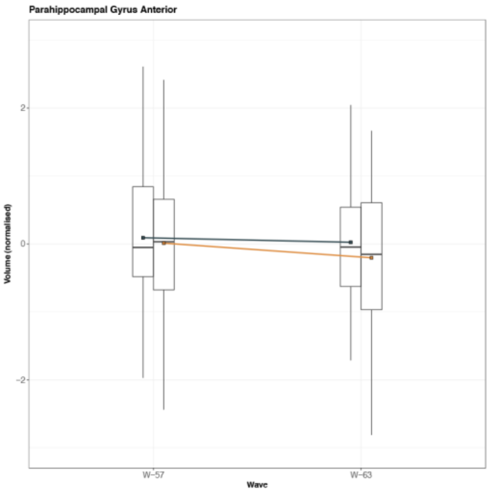
Parahippocampal Gyrus Anterior
Volume (normalised)
2
0
−2
W−57
W−63
Wave

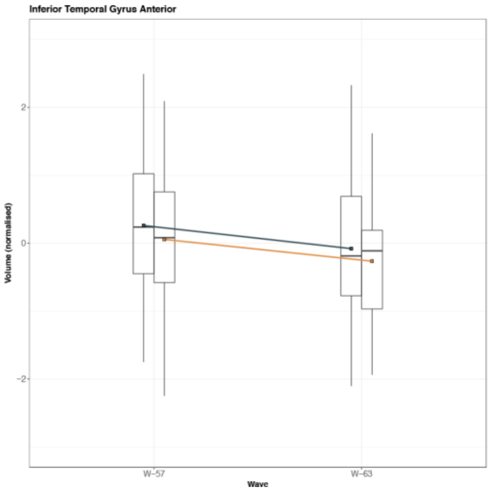
Inferior Temporal Gyrus Anterior
Volume (normalised)
2
0
−2
W−57
W−63
Wave

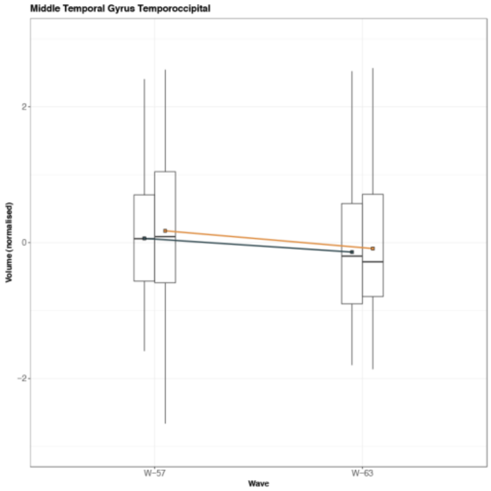
Middle Temporal Gyrus Temporoccipital
Volume (normalised)
2
0
−2
W−57
W−63
Wave

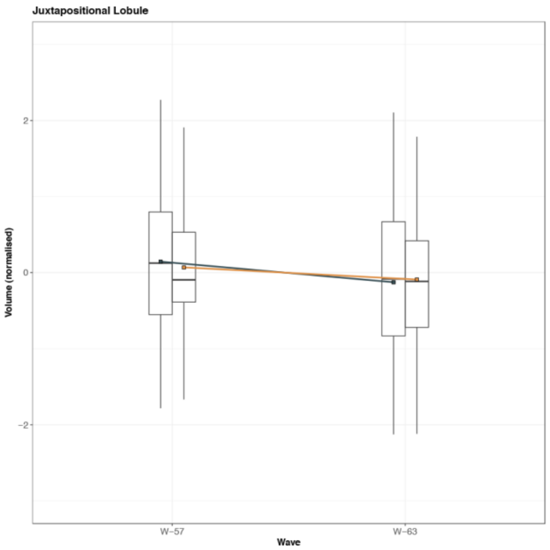

Supplementary Fig*ure* 2. Box-plot analysis showing group differences in the distribution of volume of (normalized) imaging derived phenotypes (IDPs) from ages 57yrs (W-57) to 63yrs (W-63), with group membership identified by colour. Longitudinal change in normalized volumes are shown for global brain matter (grey matter and white matter combined), total grey matter (GM), total white matter (WM), cerebral spinal fluid (CSF), and in a subset of GM volume of regions-of-interest examined. Group A = improvers (grey), Group B = decliners (orange), as identified by the extreme group design.

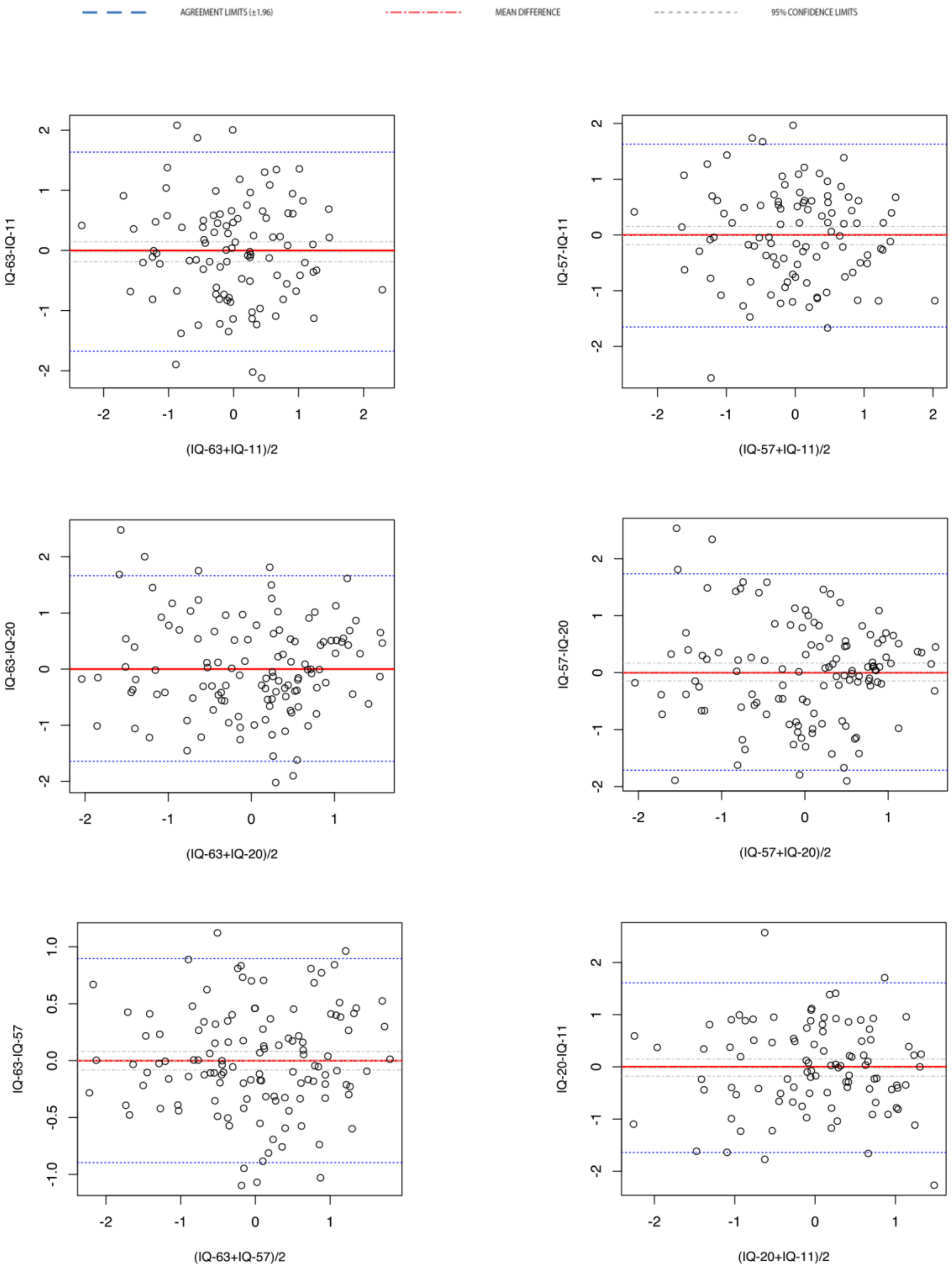

Supplementary Fig*ure* 3. Bland-Altman "limits of agreement" plot. Fig*ure* 3 displays the differences between IQ scores acquired at 4 independent measurement periods (y-axis) plotted against the average of the two scores (x-axis). The BA plots show low agreement between the IQ tests used to assess general cognitive ability across time. Additionally, the plots also indicate that the direction and magnitude of change in IQ vs initial (mean) cognitive ability are unrelated in our sample. Abbreviations: IQ-11 = IQ score at ~11 years, IQ-20 = IQ score at ~20 years, IQ-57 = IQ score ~57 years, and IQ-63 = IQ score at ~63 years.
